# Efficient Synthesis, Structural Characterization, Antibacterial Assessment, ADME-Tox Analysis, Molecular Docking and Molecular Dynamics Simulations of New Functionalized Isoxazoles

**DOI:** 10.3390/molecules29143366

**Published:** 2024-07-17

**Authors:** Aziz Arzine, Hanine Hadni, Khalid Boujdi, Khalid Chebbac, Najoua Barghady, Yassine Rhazi, Mohammed Chalkha, Asmae Nakkabi, Karim Chkirate, Joel T. Mague, Sarkar M. A. Kawsar, Ghali Al Houari, Mohammed M. Alanazi, Mohamed El Yazidi

**Affiliations:** 1 Engineering Laboratory of Organometallic, Molecular Materials and Environment, Faculty of Sciences Dhar EL Mahraz, Sidi Mohamed Ben Abdellah University, P.O. Box 1796, Atlas, Fez 30000, Morocco; arzineaziz@gmail.com (A.A.); najoua.barghady@usmba.ac.ma (N.B.); rhazifilali@gmail.com (Y.R.); asmaenakkabi@yahoo.fr (A.N.); ghalialhouari@gmail.com (G.A.H.); elyazidimohamed@hotmail.com (M.E.Y.); 2 LIMAS, Faculty of Sciences Dhar El Mahraz, Sidi Mohamed Ben Abdellah University, P.O. Box 1796, Atlas, Fez 30000, Morocco; hadni.hanine@yahoo.fr; 3 Faculty of Health and Life Sciences, INTI International University, Persiaran Perdana BBN, Putra Nilai, Nilai 71800, Malaysia; 4 Faculty of Sciences and Technologies Mohammedia, University Hassan II, B.P. 146, Mohammedia 28800, Morocco; kh.boujdi@gmail.com; 5 Laboratory of Biotechnology Conservation and Valorisation of Natural Resources, Faculty of Sciences Dhar El Mahraz, Sidi Mohamed Ben Abdallah University, Fez 30000, Morocco; khalid.chebbac@usmba.ac.ma; 6 Laboratory of Materials Engineering for the Environment and Natural Ressources, Faculty of Sciences and Techniques, University of Moulay Ismail of Meknès, B.P 509, Boutalamine, Errachidia 52000, Morocco; 7 Laboratory of Heterocyclic Organic Chemistry URAC 21, Pharmacochemistry Competence Center, Av. Ibn Battouta, BP 1014, Faculty of Sciences, Mohammed V University in Rabat, Rabat 10010, Morocco; k.chkirate@um5r.ac.ma; 8 Department of Chemistry, Tulane University, New Orleans, LA 70118, USA; joelt@tulane.edu; 9 Laboratory of Carbohydrate and Nucleoside Chemistry (LCNC), Department of Chemistry, Faculty of Science, University of Chittagong, Chittagong 4331, Bangladesh; akawsarabe@yahoo.com; 10 Department of Pharmaceutical Chemistry, College of Pharmacy, King Saud University, Riyadh 11451, Saudi Arabia; mmalanazi@ksu.edu.sa

**Keywords:** isoxazole-ester conjugates, crystal structure, antibacterial activity, docking, MD simulation, ADME-Tox

## Abstract

This work describes the synthesis, characterization, and in vitro and in silico evaluation of the biological activity of new functionalized isoxazole derivatives. The structures of all new compounds were analyzed by IR and NMR spectroscopy. The structures of **4c** and **4f** were further confirmed by single crystal X-ray and their compositions unambiguously determined by mass spectrometry (MS). The antibacterial effect of the isoxazoles was assessed in vitro against *Escherichia coli*, *Bacillus subtilis,* and *Staphylococcus* *aureus* bacterial strains. Isoxazole **4a** showed significant activity against *E. coli* and *B. subtilis* compared to the reference antibiotic drugs while **4d** and **4f** also exhibited some antibacterial effects. The molecular docking results indicate that the synthesized compounds exhibit strong interactions with the target proteins. Specifically, **4a** displayed a better affinity for *E. coli*, *S. aureus*, and *B. subtilis* in comparison to the reference drugs. The molecular dynamics simulations performed on **4a** strongly support the stability of the ligand–receptor complex when interacting with the active sites of proteins from *E. coli*, *S. aureus*, and *B. subtilis*. Lastly, the results of the Absorption, Distribution, Metabolism, Excretion and Toxicity Analysis (ADME-Tox) reveal that the molecules have promising pharmacokinetic properties, suggesting favorable druglike properties and potential therapeutic agents.

## 1. Introduction

Pathogenic microorganisms like bacteria are responsible for many serious infections, threatening well-being and even leading to large-scale health crises [1,2]. Combating these bacterial infections has become a serious and persistent concern for the global scientific community, due to the increased resistance of microorganisms caused by random genetic mutations [3,4]. Moreover, the widespread and inappropriate use of existing antibacterial drugs contributes to the proliferation of microorganisms that are resistant to antibacterial treatments [5,6]. This multi-drug resistance poses a major risk, leading to high morbidity and mortality rates [7,8]. Therefore, there is an urgent need to discover more effective therapies and develop new powerful antibacterial drugs, capable of effectively targeting a wide range of microorganisms.

Heterocyclic compounds indeed offer a rich reservoir of molecular structures with diverse biological activities, making them valuable in drug discovery to combat infectious diseases [9]. Medicinal chemists continue to focus on the modification of heterocyclic molecules to develop new therapeutic agents with improved efficacy and safety profiles [10,11]. Additionally, heterocyclic chemistry in recent years has undergone considerable development due to the diverse biological activities exhibited by many heterocyclic compounds [12,13]. The distinctive features of heterocyclic motifs lie in their structures in saturated, partially saturated, or aromatic heterocyclic compounds, as well as in functional groups [14,15]. They are the active ingredient of many families of both natural and synthetic products effective in therapeutic, agrochemical [16,17], industrial [18], and other fields.

**Figure 1:**
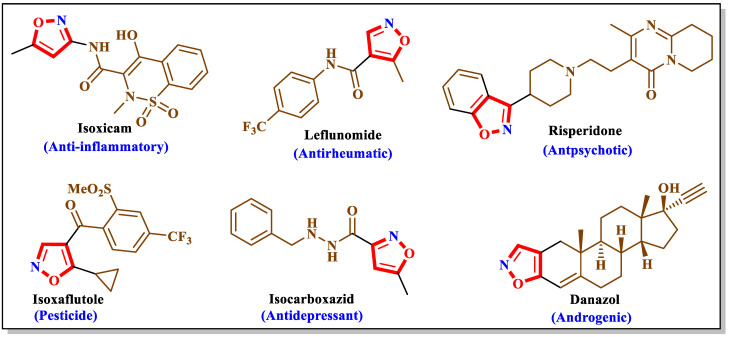
Examples of isoxazole-containing drugs.

Isoxazoles are an attractive type of heterocyclic compound for medicinal research due to their broad spectrum of biological activity. These include ones having antihistaminic [19], antifungal [20,21], antimicrobial, [22,23], antiviral [24,25], anti-inflammatory [26,27], antioxidant [22,28], and anticancer [29,30] activities as well as ones used as herbicides [31], fungicides [32], insecticides [33], and anticorrosive [34,35,36] coatings, among others. Indeed, there are several drugs currently on the market which incorporate the isoxazole moiety as the main pharmacophore (Figure 1). Also, isoxazoles are precursors to many bioactive molecules, including amino acids, amino-alcohols, and amino esters [23,24].

Chemoinformatics has indeed emerged as a crucial tool in various scientific disciplines, including chemistry, biology, and materials science [35,36]. These techniques have been widely used to discover new candidates because they offer a powerful alternative for predicting and interpreting complex experimental data. In particular, in silico molecular docking studies are of great importance in drug design, as they can predict the likely interactions between potential drug molecules (ligands) and their target proteins (enzymes or receptors) [37,38]. Moreover, molecular dynamics simulation analysis allows researchers to track the behavior of complexes formed between ligands and target proteins under in silico physiological conditions, exploring and evaluating their stability over time [39,40]. These computational simulations provide valuable insights that complement experimental methods and enhance the efficiency of the drug development process.

Building upon the observed beneficial effects demonstrated by the above-mentioned type of isoxazole compounds and as a continuation of our research on the synthesis and biological evaluation of new heterocyclic systems [41,42,43], we report here the functionalization of new isoxazole systems synthesized from (Z)-2-benzylidenebenzofuran-3(*2H*)-one (aurone) through the benzoyloxy and acetoxy groups, as well as an assessment of their antibacterial activity using both in vitro and in silico approaches.

## 2. Results and Discussion

### 2.1. Syntheses of Functionalized Isoxazoles ***4*** and ***5***

**Scheme 1:**
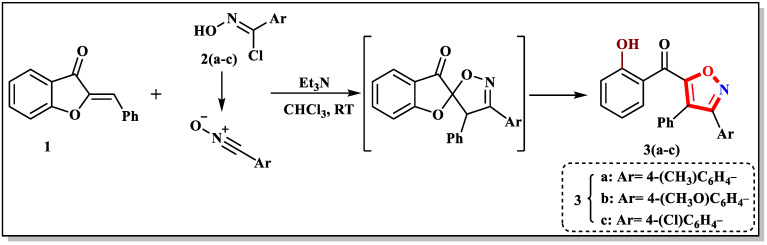
Synthetic route of the compounds **3**(**a**–**c**).

Initially, the precursors **3**(**a**–**c**) were synthesized by a 1,3-dipolar cycloaddition (1,3-DC) reaction between the dipolarophile (Z)-2-benzylidenebenzofuran-3(*2H*)-one **1** and 1,3-dipoles of the arylnitriloxide type (Scheme 1). These were generated in situ from chlorinated aldoximes **2**(**a**–**c**) and triethylamine in chloroform at ambient temperature in the presence of **1** to prevent the dimerization of the dipole into furoxane. This reaction results in the formation of isoxazoles **3**(**a**–**c**) directly instead of spiroisoxazolines [44,45].

**Scheme 2:**
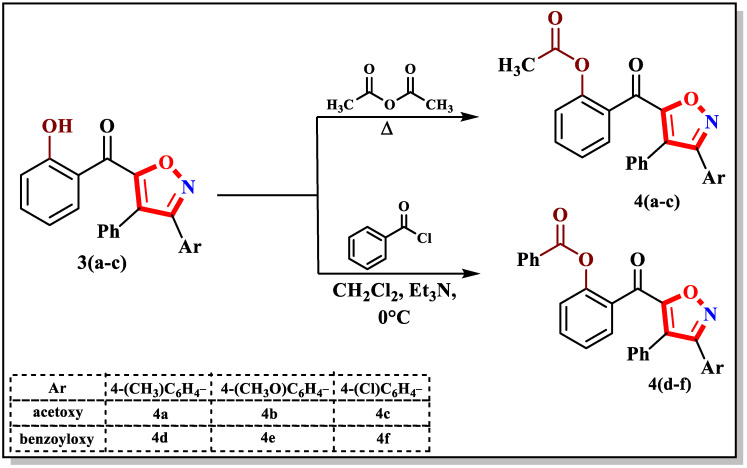
Synthetic route of functionalized isoxazole compounds.

The synthesis of the functionalized isoxazoles **4**(**a**–**f**) relied on the reactivity of the hydroxy group of **3**(**a**–**c**) towards benzoyl chloride and acetic anhydride, respectively, forming the new isoxazole compounds with ester functional group in satisfactory yields (Scheme 2).

The structures of the functionalized isoxazole products were determined by IR, ^1^H-NMR, and ^13^C-NMR spectroscopies plus MS and confirmed using single-crystal X-ray diffraction for **4c** and **4f**. The physical and spectroscopic features of all isoxazole products are outlined in Table 1. indicating the successful formation of pure products in good yields. Specifically, the IR spectra of **4**(**a**–**f**) show the disappearance of the absorption band of the alcohol function (OH) of **3**(**a**–**c**) and the appearance of an absorption band around 1750 cm^−1^ corresponding to the C=O stretching vibration of the ester group. The ^1^H-NMR spectra all indicate the disappearance of the singlet signal at 11.63 ppm assigned to the hydroxyl proton for **3**(**a**–**c**). The methyl group of the ester functional group, where present, appears as a singlet at around 2.2 ppm while the ^13^C-NMR spectra show, besides the peaks assigned to the aromatic carbons, a peak around 168 ppm attributable to the carbonyl C=O of the ester functional group. High-resolution mass spectroscopy showed molecular ion peaks exactly corresponding to the molecular masses of the proposed products.

### 2.2. X-ray Diffraction Data and Structural Determination of ***4c*** and ***4f***

**Figure 2:**
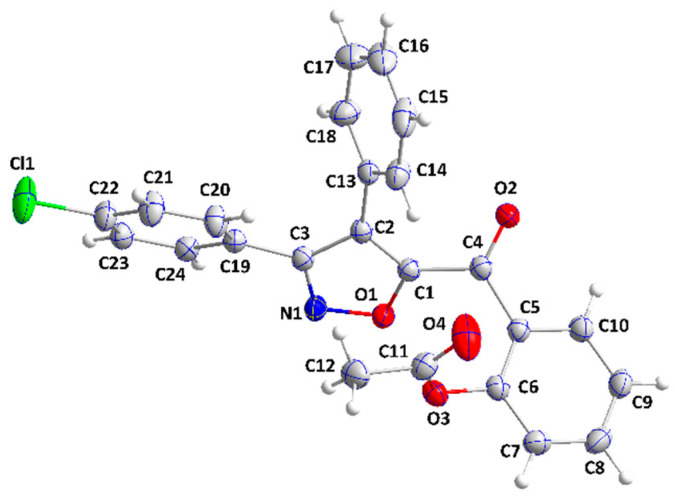
Perspective view of isoxazole **4c** with labeling scheme and 50% probability ellipsoids.

In **4c**, both substituents on the C5···C10 ring extend in the same direction, giving the molecule a “pincer-like” conformation (Figure 2).

The isoxazole ring is planar to within 0.0080(8)Å and the mean planes of the C5···C10 and the C13···C18 rings are inclined to the above plane by 62.39(5) and 59.44(6)°, respectively. The dihedral angle between the mean planes of the C19···C24 and the isoxazole rings is 35.61(4)° while that between the mean planes of the C13···C18 and the C19···C24 rings is 67.20(6)°. All the bond distances and interbond angles appear as expected for the formulation given. In the crystal, the C23—H23···O2 hydrogen bonds (Table 2) form chains of molecules that extend along the *c*-axis direction.

**Figure 3:**
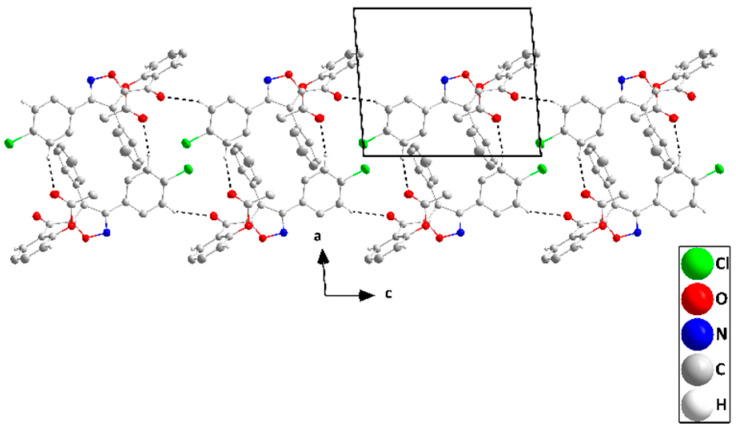
A portion of one ribbon viewed along the b-axis direction with C—H···O hydrogen bonds depicted by dashed lines. Non-interacting hydrogen atoms are omitted for clarity (isoxazole **4c**).

**Figure 4:**
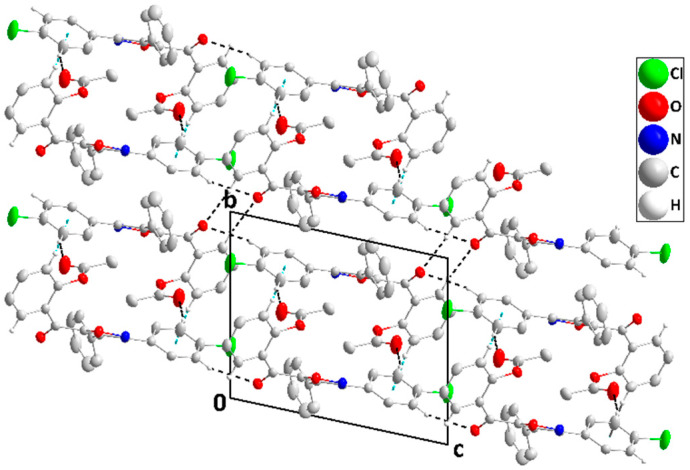
Packing viewed along the a-axis direction with C—H···O hydrogen bonds and C—H···π(ring) interactions shown, respectively, by black and light blue dashed lines. Non-interacting hydrogen atoms are omitted for clarity (isoxazole **4c**).

These are connected in pairs across inversion centers by C21—H21···O4 hydrogen bonds (Table 2) to form ribbons (Figure 3), which are then linked by C10—H10···O2 hydrogen bonds (Table 2) into stepped layers parallel to $\left(1\bar{1}0\right)$. The layers are joined by C7—H7···Cg4 interactions (Table 2) to form the full 3-D structure (Figure 4).

**Figure 5:**
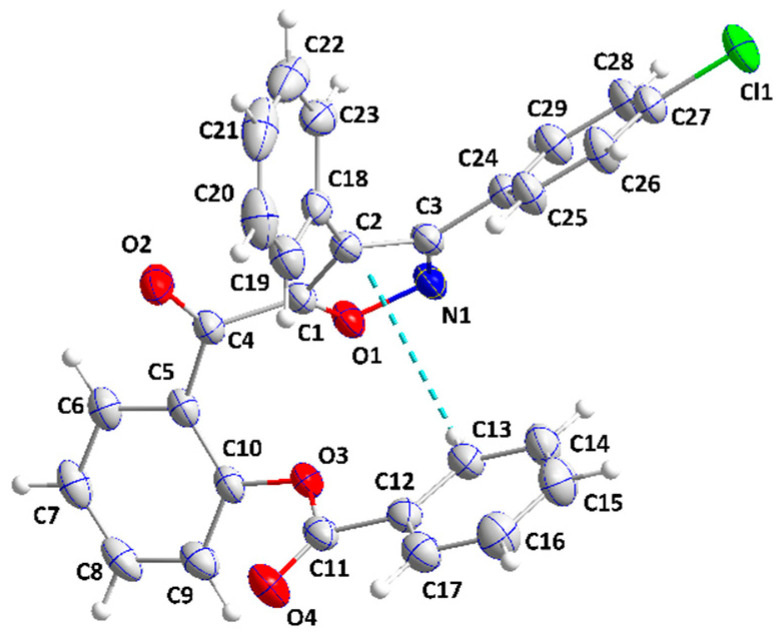
Perspective view of isoxazole **4f** with labeling scheme and 50% probability ellipsoids. The intramolecular C—H···π(ring) interaction is depicted by a dashed line.

Compound **4f** adopts a “pincer”-like conformation aided by a C13—H13···Cg1 interaction (Table 3) and with the C5···C10 ring at the vertex (Figure 5).

**Figure 6:**
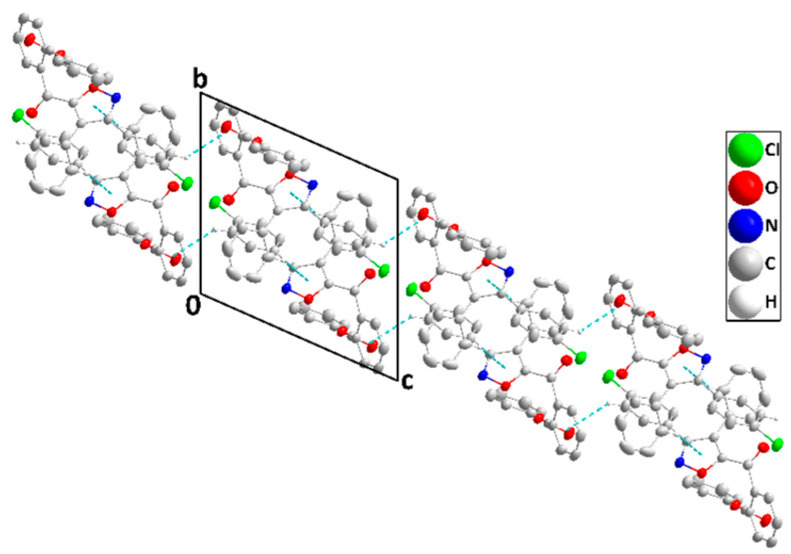
A portion of one ribbon viewed along the a-axis direction with C—H···π(ring) interactions depicted by dashed lines. Non-interacting hydrogen atoms and the intramolecular C—H···π(ring) interactions are omitted for clarity (isoxazole **4f**).

**Figure 7:**
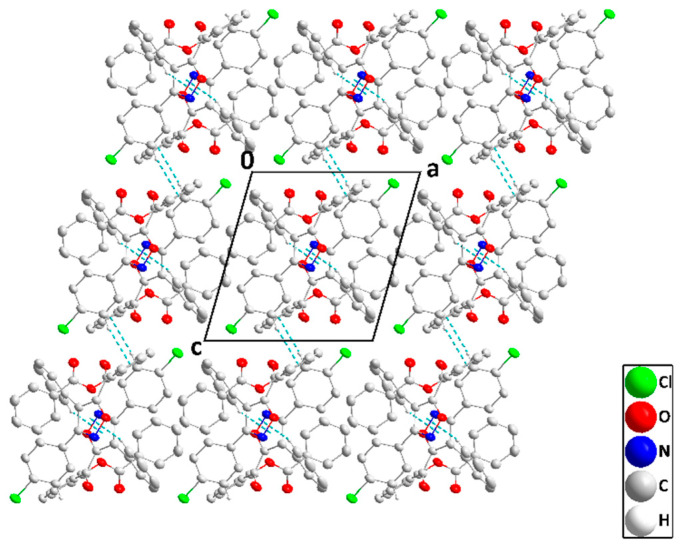
Packing viewed along the b-axis direction with C—H···π(ring) interactions depicted by dashed lines. Non-interacting hydrogen atoms and the intramolecular C—H···π(ring) interactions are omitted for clarity (isoxazole **4f**).

The isoxazole ring appears planar although the slight elongation of the displacement ellipsoids for O1 and N1 normal to the plane could suggest the presence of two opposite non-planar conformers. The mean planes of the C18···C23 and the C24···C29 rings are inclined to that of the isoxazole ring by 59.33(6) and 40.92(8)°, respectively, while the dihedral angles between the mean planes of the C5···C10 and the C12···C17 rings and that of the isoxazole ring are 64.80(7) and 55.80(7)°, respectively. All the bond distances and interbond angles appear as expected for the formulation given. In the crystal, the C28···H28···Cg2 interactions (Table 3) form chains of molecules extending along the *c*-axis direction, which are paired up by inversion-related C23—H23···Cg1 interactions (Table 3) to form ribbons (Figure 6). The ribbons pack with normal van der Waals contacts (Figure 7).

### 2.3. Antibacterial Screening of Isoxazoles ***4**(**a**–**f**)*

**Figure 8:**
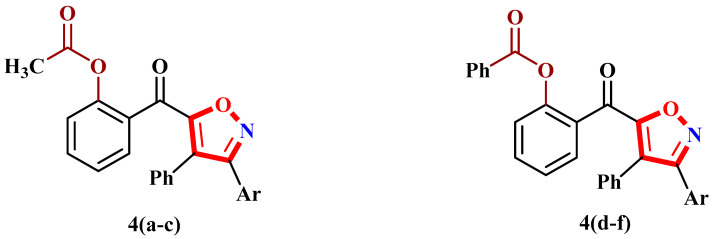
Structures of the synthesized isoxazoles **4**(**a**–**f**).

Functionalized isoxazoles play a significant role in medicinal chemistry and biological sciences and have shown many applications in drug development for the management of a variety of life-threatening pathologies [46,47]. The acetoxy **4**(**a**–**c**) and the benzoyloxy **4**(**d**–**f**) isoxazole derivatives were synthesized (Figure 8) and subsequently tested in vitro for their antibacterial action against Gram-positive and Gram-negative bacterial species such as *Staphylococcus aureus* (CECT 976), *Bacillus subtilis* (DSM 6633), and *Escherichia coli* (K12). The disk diffusion technique was used to assess bacterial susceptibility to the different isoxazoles and the results for the new isoxazoles and reference drugs are given in Table 4.

The preliminary screening results show that **4**(**a**–**f**) possess variable antibacterial properties against the tested bacteria when compared to standard drugs. The most promising results against the Gram-negative bacterium *Escherichia coli* were shown by **4a**, which carries the methyl substituent, with a zone of inhibition of 17.5 ± 1.20 mm, compared to the known antibiotic streptomycin (ZI = 24 ± 1.60). Compound **4e** showed robust efficacy against *Escherichia coli* with an inhibition zone of 14.5 ± 0.90 mm, while **4c** and **4f**, both with a chlorine substituent, showed inhibition zones of 13.5 ± 1.50 and 13.25 ± 0.40 mm, respectively. The Gram-positive bacterium *Bacillus subtilis* was found to be less sensitive to **4**(**a**–**f**). In particular, **4a** exhibited good antibacterial action against *Bacillus subtilis*, with an inhibition zone of 14.5 ± 0.80 mm, comparable to ampicillin (ZI = 16 ± 1.30 mm) while **4c**, **4d**, and **4f** also displayed antibacterial action against *Bacillus subtilis* with inhibition zones of 11 ± 1.20, 11.5 ± 1.15, and 11.25 ± 0.75 mm, respectively. For the Gram-positive bacterium strain *Staphylococcus aureus*, the best antibacterial activity was shown by **4f**, which carries the chlorine substituent, with an inhibition zone of 15.5 ± 1.55 mm, compared to ampicillin (ZI = 23 ± 2.60 mm). Compounds **4d** and **4e** also exhibited excellent action against *Staphylococcus aureus*, with inhibition zones of 12 ± 1.05 and 13.75 ± 1.25 mm, respectively. To validate the outcomes of the experimentally determined biological properties, in silico studies has been conducted.

### 2.4. Molecular Docking Studies

The molecular docking technique is used to explore how drugs bind to the active sites of target proteins, and to identify the precise interactions involved. In this context, we carried out molecular docking simulations of **4**(**a**–**f**) and proteins from different bacteria: *Escherichia coli* (**6kzv**), *Staphylococcus aureus* (**5tw8**) and *Bacillus subtilis* (**1of0**). Using these simulations, we were able to detect how these molecules bind and interact with bacterial proteins. The binding affinity results are presented in Table 5. The binding affinity observed for *E. coli* ranges from −9.38 to −12.84 kcal/mol, with streptomycin exhibiting the highest affinity. These high binding affinity values suggest that these molecules have considerable potential to act as potent inhibitors of the Escherichia coli protein receptor. In the case of *Staphylococcus aureus*, the binding affinity values range from −8.16 to −10.45 kcal/mol. Compound **4d** exhibits the highest binding affinity, which surpasses that of the standard drug Ampicillin, suggesting its good potential as an antibacterial agent against *Staphylococcus aureus*. For *Bacillus subtilis*, the range of binding affinities is −8.58 to −11.17 kcal/mol, with **4f** displaying the highest affinity, showing that **4f** outperforms Ampicillin and indicating its promising inhibitory activity against this pathogen, thereby offering a promising alternative for addressing antibacterial resistance issues associated with this strain. Furthermore, **4b** exhibits the lowest binding affinity among the tested compounds for both *Staphylococcus aureus* and *Bacillus subtilis*, which is consistent with the in vitro results. This correlation between the in silico and in vitro results reinforces the predictive value of molecular docking studies in the identification and optimization of new antibacterial agents.

**Figure 9:**
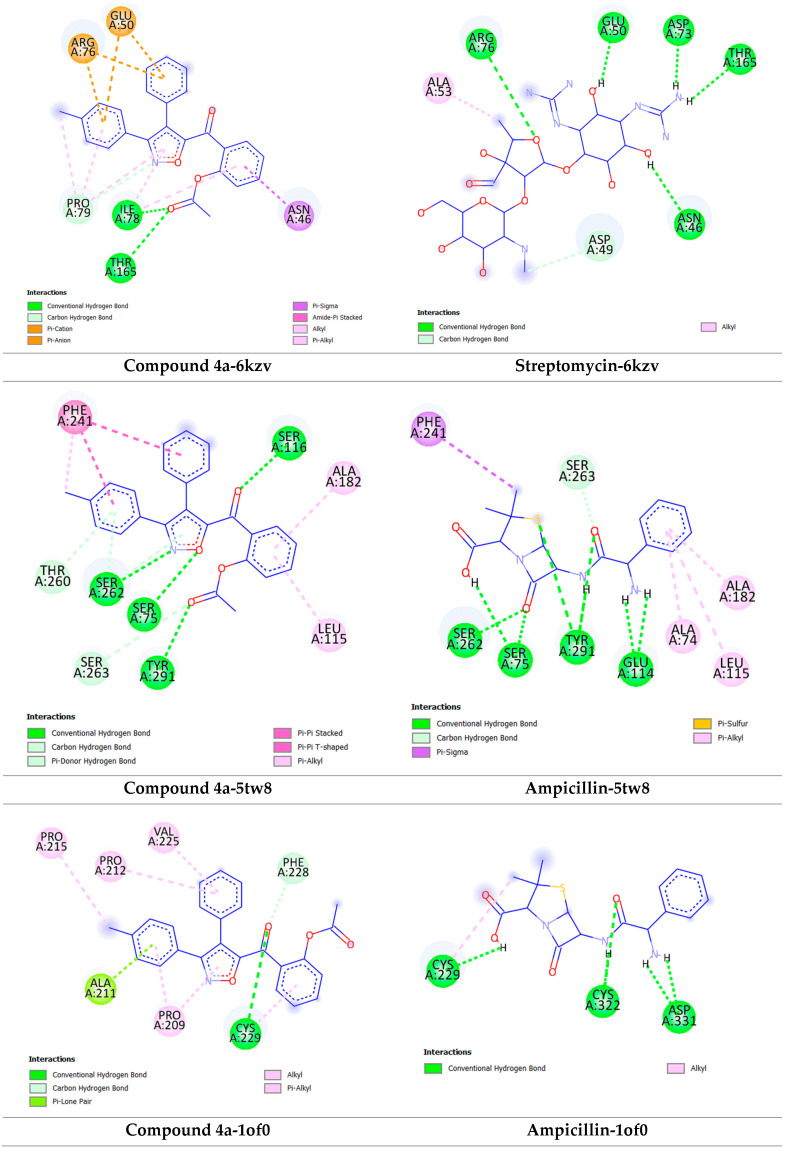
Two-dimensional visualization of the interactions between **4a** and antibacterial proteins versus reference drugs.

The energetics of the interaction of **4**(**a–f**) associated with docking revealed a similar complexation energy for the majority of them, indicating that the molecular interactions are quite similar in all cases. To elucidate these interactions, a detailed study was carried out on **4a** with the aim of identifying the essential interactions between **4a** and the target proteins of different bacteria and to assess its potential as a multi-target inhibitor. The results show that **4a** forms specific interactions with proteins from *Escherichia coli*, *Staphylococcus aureus* and *Bacillus subtilis* (shown in Figure 9) which are similar to those of the reference drugs.

For *Escherichia coli* (6kzv), **4a** formed two hydrogen bonds with the residues ILE78 (2.76 Å) and THR165 (2.83 Å), two Pi-Cation electrostatic interactions with ARG76 (3.96 Å and 3.79 Å), and two Pi-Anion interactions with GLU50 (3.36 Å and 4.076 Å). On the other hand, streptomycin forms hydrogen bonds with ASN46, GLU50, ASP73, ARG76, and THR165, at distances of 2.81 Å, 1.86 Å, 1.72 Å, 2.75 Å, and 2.25 Å, respectively. For *Staphylococcus aureus*, **4a** formed four significant hydrogen bonds with SER75, SER116, SER262, and TYR291, at distances of 2.48 Å, 2.59 Å, 2.83 Å, and 2.89 Å, respectively, showing similar interactions compared to ampicillin, which interacts through several hydrogen bonds with SER75 (2.81 Å and 2.15 Å), GLU114 (2.21 Å and 2.06 Å), SER262 (2.23 Å), and TYR291 (2.86 Å and 2.44 Å). Finally, for *Bacillus subtilis*, **4a** formed a significant hydrogen bond-type interaction with CYS229 (2.19 Å). Conversely, ampicillin established several hydrogen bonds with CYS229 (2.39 Å), CYS322 (1.85 Å and 2.20 Å), and ASP331 (1.88 Å and 1.75 Å), illustrating increased complexity in its interaction with the bacterial target.

### 2.5. Molecular Dynamics Simulation Analysis

**Figure 10:**
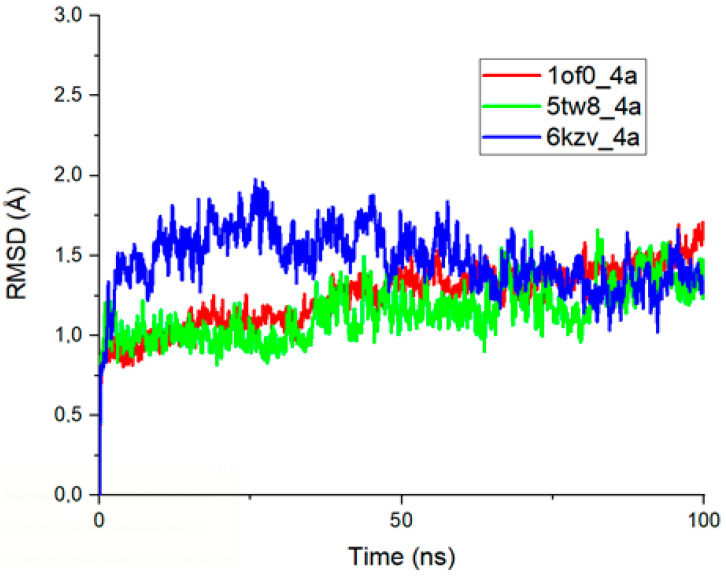
The RMSD values of *Escherichia coli* (**6kzv_4a**), *Staphylococcus aureus* (**5tw8_4a**), and *Bacillus subtilis* (**1of0_4a**) in complex with **4a**.

**Figure 11:**
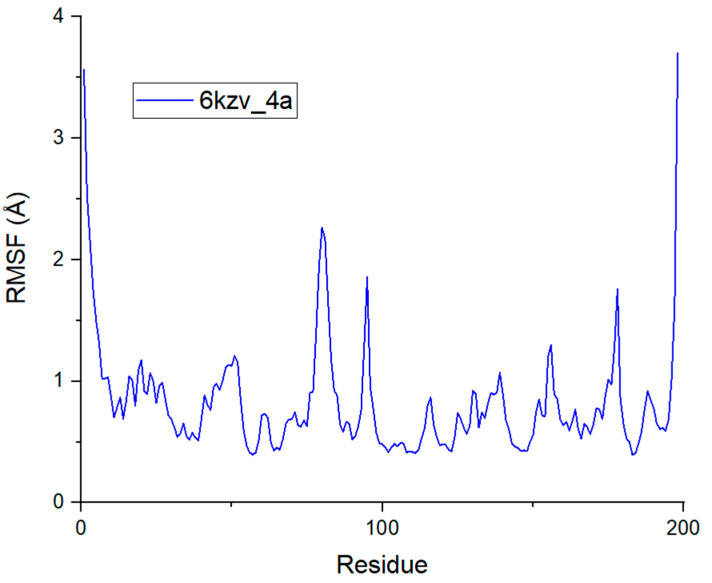

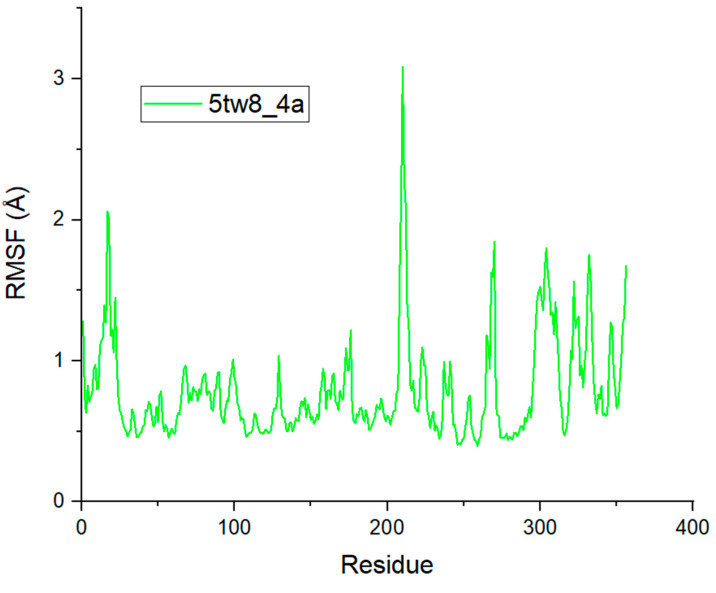

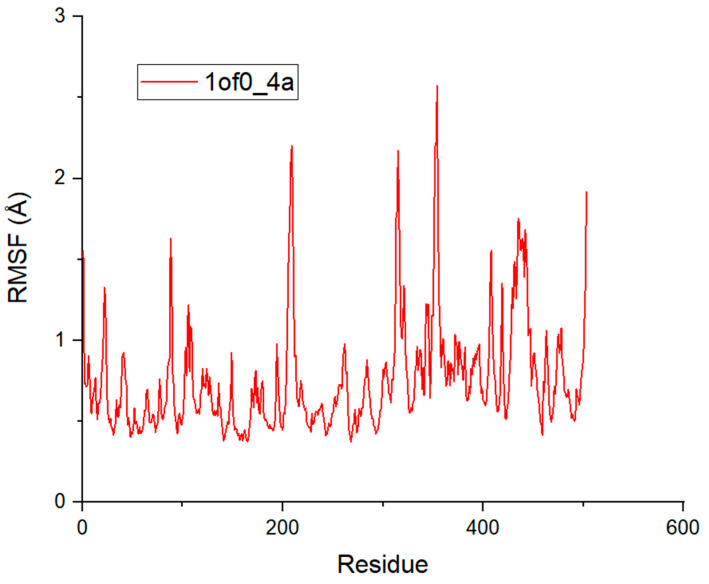
The RMSF values of *Escherichia coli* (**6kzv_4a**), *Staphylococcus aureus* (**5tw8_4a**), and *Bacillus subtilis* (**1of0_4a**) in complex with **4a**.

Since **4a** was identified to have the best antibacterial efficacy, the molecular docking results were supplemented by molecular dynamics (MD) simulations. Variations in the conformation and dynamics of the complexes formed between *Escherichia coli*, *Bacillus subtilis*, *Staphylococcus aureus,* and **4a** were quantified using the RMSD (root mean square deviation) and RMSF (root mean square fluctuation) indicators, illustrated in Figure 10 and Figure 11, respectively.

In the case of the *Escherichia coli* (6kzv) protein (Figure 10), the RMSD changes reveal a significant increase in the oscillations which rise from 0.8 to 1.6 Å in just 10 nanoseconds. This increase seems to be mainly due to the initial kinetic effect that the complexes display during the transition phase. After this phase, stabilization and equilibrium were observed for all the complexes. On the other hand, for *Staphylococcus aureus* (5tw8) and *Bacillus subtilis* (1of0), the RMSD evolutions indicate almost identical variations, with an oscillation between 1 and 1.5 Å throughout the molecular dynamics (MD) simulation. The average values (RMSD) observed for the complexes formed between **4a** and the proteins of *Escherichia coli*, *Staphylococcus aureus,* and *Bacillus subtilis* were 1.82 Å, 1.56 Å, and 1.70 Å, respectively. The fact that these RMSD values are below 2 Å means that **4a** has remarkable stability within the active sites of *Escherichia coli*, *Staphylococcus aureus,* and *Bacillus subtilis* proteins.

The analysis of the RMSF trajectories provides essential information on the stability of the ligand–receptor interaction, enabling us to assess the stability, rigidity, and compactness of the protein being analyzed. A high RMSF value indicates high flexibility, meaning that the residues are less stable, while a low value indicates greater rigidity and stability. For the protein from *Escherichia coli* (6kzv) (Figure 11), most residues display similar RMSF values, although some show notable deviations, notably at the ASP6 (3.56 Å), THR85 (2.26 Å), ALA100 (1.85 Å), GLY200 (1.76 Å), and GLY220 (3.69 Å) positions. These variations are mainly found in regions of the protein that are not directly involved in its activity, making their impact relatively low. In contrast, essential residues within the active site, such as GLU50, ASP73, ARG76, and THR165, show much more restricted movements, with RMSFs of no more than 0.9 Å, indicating the importance of the hydrogen bonds formed by these residues in stabilizing the interactions between the *Escherichia coli* (6kzv) protein and the ligands, thereby ensuring the greater stability of the complex. For the *Staphylococcus aureus* (5tw8) and *Bacillus subtilis* (1of0) proteins (Figure 3), the RMSF fluctuations indicate significant variations for certain residues; for example, ALA41 (2.05 Å), HIS234 (3.08 Å), GLY296 (1.84 Å), GLY330 (1.97 Å), and ASP358 (1.75 Å) in *Staphylococcus aureus*, and PRO217 (2.01 Å), GLY323 (2.17 Å), GLN362 (2.57 Å), and PRO511 (1.91 Å) in *Bacillus subtilis*. These residues located outside the active sites have a limited impact on the protein’s ability to bind ligands. On the other hand, residues fundamental to the function of these proteins showed low RMSF values, suggesting the existence of hydrogen bonds for stabilizing the protein–ligand complexes in *Staphylococcus aureus* and *Bacillus subtilis*. These observations confirm the results of the RMSD analysis, highlighting good structural stability in the ligand–protein complexes, mainly due to strong hydrogen bonding interactions.

### 2.6. ADME-Tox Analyses

The evaluation of **4a**, **4b**, **4c**, **4d**, **4e**, and **4f** as potential pharmaceutical agents for the treatment of bacterial infections was based on the predictions of their ADMET pharmacokinetic parameters. The results, presented in Table 6, show promising pharmacokinetic properties, suggesting their potential efficacy as therapeutic agents.

The intestinal absorption rates of the compounds ranged from 97.961% to 100%, indicating the excellent absorption capacity in the human intestine, which is crucial for their oral efficacy. With regard to distribution, the predictions concerning the permeability of the blood–brain barrier (BBB) and access to the central nervous system (CNS) indicate that all the compounds analyzed show negative values for LogBB and LogPS [48]. These results imply a limited distribution within these zones. This limitation is potentially beneficial in the treatment of certain bacterial infections, as it reduces the likelihood of neurological side effects by preventing drugs from reaching the CNS in large quantities. The metabolism studies showed that all the compounds acted as potential substrates and/or inhibitors of the CYP3A4 enzyme, which played a crucial role in this study [49,50]. The total clearance values suggest moderate to low excretion for all compounds, suggesting that these molecules could persist in the body for a prolonged period. Finally, the predicted non-toxicity of all the compounds studied is a positive aspect that reinforces the safety profile of the ligands for further development. In summary, the ADMET prediction results offer valuable insights into the potential of the synthesized compounds as future therapeutic agents.

## 3. Summary

To conclude, this work reports the synthesis, crystallographic analysis, biological evaluation, and in silico studies of new ester-functionalized isoxazole compounds. The compounds were obtained through a series of reactions using an efficient and robust procedure starting from 2-benzylidenebenzofuran-3(2H)-one (aurone). Structural determination was achieved using IR, ^1^H-NMR, and ^13^C-NMR spectroscopy and mass spectrometry with further confirmation via X-ray diffraction for compounds **4c** and **4f**. The antibacterial screening of the synthesized ester-functionalized isoxazole compounds against three bacterial strains—*Escherichia coli*, *Staphylococcus aureus*, and *Bacillus subtilis*—showed that the compounds possess good antibacterial properties. Furthermore, the molecular docking studies revealed significant binding interactions between the isoxazole compounds and the bacterial target proteins of *Escherichia coli* (**6kzv**), *Staphylococcus aureus* (**5tw8**), and *Bacillus subtilis* (**1of0**). The MD simulation results indicate the remarkable stability of compound 4a within the active sites of *Escherichia coli*, *Staphylococcus aureus,* and *Bacillus subtilis* proteins. Lastly, the results of the ADMET profiles of the isoxazole compounds indicate that they have good bioavailability. Taken together, the findings of this research could provide important insights to design and synthesize new ester-functionalized isoxazole compounds that are likely to be promising antibacterial agents.

## 4. Materials and Methods

### 4.1. Chemistry

Complete insights into the reagents and solvents required for compound synthesis and the equipment used in spectroscopic characterization and X-ray crystallographic analysis as well as the relevant synthesis procedures are clearly described in the Supplementary Material file.

### 4.2. Antibacterial Screening Protocol

The technique and bacterial strains used to screen the antibacterial properties of **4**(**a**–**f**) are included in the Supplementary Material file.

### 4.3. Molecular Docking

We conducted molecular docking simulations with compounds **4**(**a**–**f**) using specific target proteins from different bacteria. The proteins selected for these simulations are as follows:

➢ *Escherichia coli* **(PDB ID: 6kzv)** [51]:
  - Target protein: Gyrase A (DNA gyrase subunit A).
  - Biological role: Gyrase A is essential for bacterial survival as it catalyzes the negative supercoiling of DNA, which is crucial for DNA replication and transcription. Inhibiting this enzyme can prevent DNA replication, leading to cell death.
➢ *Staphylococcus aureus* **(PDB ID: 5tw8)** [52]:
  - Target protein: PBP2a (Penicillin-binding protein 2a).
  - Biological role: PBP2a is involved in bacterial cell wall synthesis. This protein confers resistance to β-lactam antibiotics by preventing these antibiotics from binding to penicillin-binding proteins, allowing the bacteria to continue synthesizing its cell wall despite the presence of the antibiotic.
➢ *Bacillus subtilis* **(PDB ID: 1of0)** [53]:
  - Target protein: endospore coat protein.
  - Biological role: This protein is involved in the formation of the endospore coat, a resistant structure that protects bacterial spores under extreme environmental conditions. Inhibiting this protein can disrupt spore formation, reducing bacterial survival in adverse conditions.

Three-dimensional crystal structures of target proteins were extracted from the Protein Data Bank (PDB). These structures were prepared by removing water molecules, ligands, and non-protein elements. The software tools Discovery Studio, Autodock 4 and Autodock Tools were used to further investigate compound–protein interactions and evaluate interaction energies using a three-dimensional grid [54]. The dimensions of the central analysis grid were carefully adjusted for each protein to ensure accurate placement of ligands in the complex [44,53]. The results of the study were visualized in two dimensions to better understand the binding interactions.

### 4.4. Molecular Dynamics (MD)

Molecular dynamics simulations used NAMD software [55] associated with CHARMM36 force field [56]. The simulation setup consisted of enclosing the system in a cubic box, measuring 10 Å on each side, filled with TIP3P water molecules. The system was neutralized by adding NaCl ions at a concentration of 0.154 M, and applying the Monte Carlo technique [57]. The initial phase involved minimizing the energy of each system using a 10,000-step gradient descent method. This was followed by a 100 ns stabilization phase under the NVT ensemble (number of particles, volume, temperature), keeping the system at a constant temperature of 310 K. This was followed by an additional 100 ns of unrestricted molecular dynamics simulation under the NPT (number of atoms, pressure, temperature) ensemble for each system [48]. The stability of the systems was evaluated by analyzing the molecular dynamics trajectories through the Visual Molecular Dynamics (VMD) software [58].

### 4.5. In Silico Pharmacokinetics ADMET

The integration of computer technology has transformed the way we discover new drugs, making the process both faster and more accurate [58]. Computational studies give us an improved understanding of a compound’s behavior with regard to absorption, distribution, metabolism, excretion, and toxicity (ADMET). These analyses exploit information on how a drug acts to anticipate its effects from the earliest stages of development. The pkCSM online platform is particularly useful for assessing a drug’s capacity to be absorbed by the human intestine, its distribution in the body, its biological transformation and elimination, as well as its toxicity profile [56]. Computer simulation has thus become indispensable in the evaluation of pharmacokinetic parameters linked to ADMET.

## Figures and Tables

**Table 1:** Physicochemical characteristics and characterization data of **4**(**a**–**f**).

N°	Ar	Formula (M. g/mol)	M.p (°C)	Yield ^a^ (%)	^1^H-NMR (ppm)	^13^C-NMR (ppm)	IR (cm^−1^)	MS *(m/z)* [M+H]^+^
					CH_3_	C=O_(ketone)_	C=O_(ketone)_	
					OCH_3_	C=O_(ester)_	C=O_(ester)_	
					CH_3(ester)_	C=N	C=N	
**4a**	4-(CH_3_)C_6_H_4_	C_25_H_19_NO_4_ (397.43)	142–144	95	3.38	181.78	1667	398.41
					---	168.91	1753	
					2.20	162.30	1603	
**4b**	4-(CH_3_O)C_6_H_4_	C_25_H_19_NO_5_ (413.42)	140–142	90	---	181.79	1666	414.36
					3.83	168.92	1756	
					2.20	160.92	1604	
**4c**	4-(Cl)C_6_H_4_	C_24_H_16_ClNO_4_ (417.84)	128–130	85	---	181.55	1677	417.34
					---	168.95	1764	
					2.21	161.39	1609	
**4d**	4-(CH_3_)C_6_H_4_	C_30_H_21_NO_4_ (459.50)	118–120	92	2.23	182.07	1667	461.42
					----	164.36	1754	
					----	161.89	1600	
**4e**	4-(CH_3_O)C_6_H_4_	C_30_H_21_NO_5_ (475.50)	124–126	89	----	182.08	1670	476.43
					3.9	164.35	1741	
					----	161.99	1597	
**4f**	4-(Cl)C_6_H_4_	C_29_H_18_ClNO_4_ (479.91)	126–130	91	-----	181.88	1677	481.36
					-----	164.30	1760	
					-----	161.39	1609	

^a^ Isolated yield of products after purification.

**Table 2:** Hydrogen bond geometry (Å, °). Cg4 is the centroid of the C19···C24 benzene ring.

D—H···*A*	*D*—H	H···*A*	*D*···*A*	*D*—H···*A*
C7—H7···Cg4 ^i^	0.95	2.77	3.5994 (17)	146
C10—H10···O2 ^ii^	0.95	2.43	3.3645 (18)	170
C21—H21···O4 ^iii^	0.95	2.36	3.289 (2)	164
C23—H23···O2 ^iv^	0.95	2.58	3.4615 (17)	154

Symmetry codes: (i) −*x* + 1, −*y* + 1, −*z* + 1; (ii) −*x* + 1, −*y* + 2, −*z* + 2; (iii) −*x*, −*y* + 1, −*z* + 1; (iv) *x*, *y*, *z* − 1.

**Table 3:** Cg1 and Cg2 are, respectively, the centroids of the C1/C2/C3/O1/N1 and the C5···C10 rings.

*D*—H···*A*	*D*—H	H···*A*	*D*···*A*	*D*—H···*A*
C13—H13···Cg1	0.95	2.87	3.534 (2)	128
C23—H23···Cg1 ^i^	0.95	2.87	3.744 (2)	154
C28—H28···Cg2 ^ii^	0.95	2.76	3.532 (2)	140

Symmetry codes: (i) −*x* + 1, −*y* + 1, −*z* + 1; (ii) *x*, *y*, *z* − 1.

**Table 4:** In vitro antibacterial activity of **4**(**a**–**f**).

Zone of Inhibition (ZI) in mm ^a^					
Compounds			Tested Bacteria		
N°	Ar	*Group*	*Escherichia coli*	*Bacillus subtilis*	*Staphylococcus aureus*
**4a**	4-(CH_3_)C_6_H_4_	acetoxy	17.5 ± 1.20	14.5 ± 0.80	9.5 ± 1.40
**4b**	4-(CH_3_O)C_6_H_4_	acetoxy	12.5 ± 1.40	-	-
**4c**	4-(Cl)C_6_H_4_	acetoxy	13.5 ± 1.50	11 ± 1.20	10.5 ± 1.50
**4d**	4-(CH_3_)C_6_H_4_	benzoyloxy	11 ± 0.50	11.5 ± 1.15	12 ± 1.05
**4e**	4-(CH_3_O)C_6_H_4_	benzoyloxy	14.5 ± 0.90	09 ± 0.60	13.75 ± 1.25
**4f**	4-(Cl)C_6_H_4_	benzoyloxy	13.25 ± 0.40	11.25 ± 0.75	15.5 ± 1.55
Ampicillin			NT	16 ± 1.30	23 ± 2.60
Streptomycin			24 ± 1.60	NT	NT

**^a^** Results are expressed as means ± standard deviations of duplicate experiments; NT: not tested. -: no activity.

**Table 5:** Binding affinity between **4**(**a**–**f**) and bacterial proteins (kcal/mol).

Compounds	*Escherichia coli* (6kzv) (kcal/mol)	*Staphylococcus aureus* (5tw8) (kcal/mol)	*Bacillus subtilis* (1of0) (kcal/mol)
**4a**	−10.82	−9.01	−9.21
**4b**	−10.18	−8.16	−8.58
**4c**	−9.38	−10.02	−8.75
**4d**	−10.24	−10.45	−10.47
**4e**	−10.07	−10.43	−10.32
**4f**	−10.85	−10.34	−11.17
Ampicillin		−9.58	−8.82
Streptomycin	−12.84		

**Table 6:** In silico ADMET predictions for **4a**, **4b**, **4c**, **4d**, **4e**, and **4f**.

Compounds	Absorption	Distribution		Metabolism							Excretion	Toxicity
	Intestinal Absorption (Human)	BBB Permeability	CNS Permeability	Substrate		Inhibitor					Total Clearance	AMES Toxicity
				CYP								
				2D6	3A4	1A2	2C19	2C9	2D6	3A4		
	Numeric (% Absorbed)	Numeric (LogBB)	Numeric (LogPS)	Categorical (Yes/No)							Numeric (Log mL/min/kg)	Categorical (Yes/No)
**4a**	99.42	−0.626	−1.646	No	Yes	Yes	Yes	Yes	No	Yes	0.283	No
**4b**	100	−0.873	−1.921	No	Yes	Yes	Yes	Yes	No	Yes	0.314	No
**4c**	97.961	−0.826	−1.606	No	Yes	Yes	Yes	Yes	Yes	Yes	0.15	No
**4d**	99.926	−0.637	−1.364	No	Yes	No	Yes	Yes	No	No	0.502	No
**4e**	100	−0.884	−1.639	No	Yes	No	Yes	Yes	No	Yes	0.486	No
**4f**	98.467	−0.837	−1.324	No	Yes	No	Yes	Yes	No	No	0.11	No

## Data Availability

All the data generated by this research is included in the article and Supplementary Materials.

## Supplementary Materials

The following supporting information can be downloaded at https://www.mdpi.com/article/10.3390/molecules29143366/s1, Spectroscopic data and copies of IR, NMR, and MS spectra of the newly synthesized compounds, and all additional details can be found in the electronic Supplementary Material. CCDC 2348110 and 2348111 contain the supplementary crystallographic data for the compounds **4c** and **4f**, and can be obtained free of charge from the Cambridge Crystallographic Data Centre via www.ccdc.cam.ac.uk/data_request/cif. References [46,59,60,61,62,63,64,65,66,67] are cited in the supplementary materials.
