# Supplementary material for: Efficient Synthesis, Structural Characterization, Antibacterial Assessment, ADME-Tox Analysis, Molecular Docking and Molecular Dynamics Simulations of New Functionalized Isoxazoles
Source: Molecules. 2024 Jul 17;29(14):3366. doi: 10.3390/molecules29143366 (PMC11279828; doi:10.3390/molecules29143366)
Supplement: Supplementary file 1 [file molecules-29-03366-s001.zip › molecules-3030468-supplementary.pdf]

molecules

Supplementary Information for paper

Efficient Synthesis, Structural Characterization, Antibacterial Assessment, ADME-Tox Analysis, Molecular Docking and Molecular Dynamics Simulations of New Functionalized Isoxazoles

Aziz Arzine [1], Hanine Hadni [2,3], Khalid Boujdi [4], Khalid Chebbac [5], Najoua Barghady [1], Yassine Rhazi [1], Mohammed Chalkha [1,6,*], Asmae Nakkabi [1,6], Karim Chkirate [7], Joel T. Mague [8], Sarkar M. A. Kawsar [9], Ghali Al Houari [1], Mohammed M. Alanazi [10] and Mohamed El Yazidi [1]

[1] Engineering Laboratory of Organometallic, Molecular Materials and Environment, Faculty of Sciences Dhar EL Mahraz, Sidi Mohamed Ben Abdellah University, P.O. Box 1796, Atlas, Fez 30000, Morocco; arzineaziz@gmail.com (A.A.); najoua.barghady@usmba.ac.ma (N.B.); rhazifilali@gmail.com (Y.R.); asmaenakkabi@yahoo.fr (A.N.); ghalialhouari@gmail.com (G.A.H.); elyazidimohamed@hotmail.com (M.E.Y.)
[2] LIMAS, Faculty of Sciences Dhar El Mahraz, Sidi Mohamed Ben Abdellah University, P.O. Box 1796, Atlas, Fez 30000, Morocco; hadni.hanine@yahoo.fr
[3] Faculty of Health and Life Sciences, INTI International University, Persiaran Perdana BBN, Putra Nilai, Nilai 71800, Malaysia
[4] Faculty of Sciences and Technologies Mohammedia, University Hassan II, B.P. 146, Mohammedia 28800, Morocco; kh.boujdi@gmail.com
[5] Laboratory of Biotechnology Conservation and Valorisation of Natural Resources, Faculty of Sciences Dhar El Mahraz, Sidi Mohamed Ben Abdallah University, Fez 30000, Morocco; khalid.chebbac@usmba.ac.ma
[6] Laboratory of Materials Engineering for the Environment and Natural Ressources, Faculty of Sciences and Techniques, University of Moulay Ismail of Meknès, B.P 509, Boutalamine, Errachidia 52000, Morocco
[7] Laboratory of Heterocyclic Organic Chemistry URAC 21, Pharmacochemistry Competence Center, Av. Ibn Battouta, BP 1014, Faculty of Sciences, Mohammed V University in Rabat, Rabat 10010, Morocco; k.chkirate@um5r.ac.ma
[8] Department of Chemistry, Tulane University, New Orleans, LA 70118, USA; joelt@tulane.edu
[9] Laboratory of Carbohydrate and Nucleoside Chemistry (LCNC), Department of Chemistry, Faculty of Science, University of Chittagong, Chittagong 4331, Bangladesh; akawsarabe@yahoo.com
[10] Department of Pharmaceutical Chemistry, College of Pharmacy, King Saud University, Riyadh 11451, Saudi Arabia; mmalanazi@ksu.edu.sa
* Correspondence: mohammed.chalkha1@usmba.ac.ma (Mohammed CHALKHA)

Table of contents

1. *General information* 3

2. Synthesis methods 3

   2.1 Synthesis of 5-(2-acetoxybenzoyl)-3-aryl-4-phenyl isoxazole **(4a-c)** 3

   2.2 Synthesis of 3-aryl-5-(2-benzoyloxybenzoyl)-4-phenyl isoxazole (**4d-f**) 4

3. X-ray crystallography analysis 4

4. Disk diffusion method 7

5. Characterization data 7

   5.1 5-(2-hydroxybenzoyl)-4-phenyl-3-(4-methoxyphenyl)isoxazole (**3b**): 7

   5.2 Spectral analysis of isoxazole-ester (**4a-c**) 7

   5.3 Spectral analysis of isoxazole-ester (**4d-f**) 9

6. Copies of ^{1}H, ^{13}C NMR and MS spectra of all new compounds 11

References 27

1. *General information*

All reagents and solvents were of A. R. grade. They were purchased from Sigma-Aldrich and other commercial suppliers and were used without further purification. The progress of the reactions was monitored by TLC, performed on pre-coated Merck silica gel 60 F254 plates. Column chromatography was carried out using Merck silica gel (70-230 mesh) and eluting with n-hexane and ether solution. Melting points were determined using a KOFLER Bench apparatus. ^{1}H and ^{13}C NMR spectra were recorded at room temperature on a BRUKER AVANCE II 300 Ultra-Shield (300 MHz for ^{1}H and 75 MHz for ^{13}C) spectrometer using $CDCl_3$ and DMSO-d_6 solvents. The Jmod experiment, which was employed for the ^{13}C NMR spectra, reveals information on the number of ^{13}C signals (CH_3, CH_2, CH and Cq). CH_3 and CH carbons are represented by the negative signals in these spectra, while CH_2 and C quaternary carbons are represented by the positive signals. ^{1}H and ^{13}C NMR chemical shifts (δ) are expressed in ppm referenced to the solvent used, and proton coupling constants (J) are reported in Hz. The spin multiplicities are reported as singlet (s), doublet (d), triplet (t), multiplet (m), doublet of doublets (dd), triplet of doublets (td), doublet of triplets (dt) and broad (br). Aurone **1** used as starting material in this work was synthesized following the procedures described earlier [1, 2]. The synthesis method for compounds 3a-c and characterization data of the compounds 3a and 3c were described in our previously published article [3].

2. *Synthesis methods*

2.1 *Synthesis of 5-(2-acetoxybenzoyl)-3-aryl-4-phenyl isoxazole (4a-c)*

The 3-aryl-5-(2-hydroxybenzoyl)-4-phenylisoxazole **3a-c** (1mol) and 30 ml of acetic anhydride were added to a 50 ml condenser flask. The mixture was stirred at reflux until the reaction was complete as shown by TLC. The excess acetic anhydride was then distilled off. The crude product was poured into ice-cold water (200 ml) and extracted with DCM (3 x 20 ml). The DCM layer was washed three times with a saturated solution of $NaHCO_3$ and water. The organic layer was dried over anhydrous Na_2SO_4 and the solvent was removed under vacuum. The residue obtained was purified by column chromatography using the hexane-ether mixture (3/2) and then recrystallized in ethanol.

2.2 *Synthesis of 3-aryl-5-(2-benzoyloxybenzoyl)-4-phenyl isoxazole (4d-f)*

To 20 ml of absolute dichloromethane (CH_2Cl_2), 3-aryl-5-(2-hydroxybenzoyl)-4-phenylisoxazole **3a-c** (1 mol) and triethylamine (1.2 mol) were added. The mixture was stirred at 0°C in an ice bath. Then, the benzoyl chloride (1.2 mol) was added dropwise and the progress of the reaction was monitored by TLC. When the reaction was complete, the mixture was transferred to a separating funnel and washed several times with water. The organic layer was dried over anhydrous sodium sulfate (Na_2SO_4) and the solvent was removed under reduced pressure. The residue obtained was purified by column chromatography using the hexane-ether mixture (3/2) and then recrystallized in ethanol.

3. *X-ray crystallography analysis*

Suitable crystals of **4c** and **4f** were mounted on polymer loops with a drop of heavy oil and placed in a cold nitrogen stream on the diffractometer. Intensity data were collected under control of the *APEX4* software [4] and reduced to F^2 values with *SAINT* [4] which also performed a global refinement of unit cell parameters using ≈ 9800 reflections drawn from the full data set. Application of an empirical absorption correction and merging of equivalent reflections was accomplished with *SADABS* [5] and the structures were solved by dual space methods (*SHELXT* [6]). The models were refined by full-matrix, least-squares procedures (*SHELXL* [7]) with H-atoms attached to carbon placed in calculated positions (C—H = 0.95 - 0.98 Å). All were included as riding contributions with isotropic displacement parameters 1.2 - 1.5 times those of the attached atoms. One reflection affected by the low temperature nozzle was omitted from the final refinement for **4c**.

Crystal data, data collection and structure refinement details are summarized in Table S1 and S2.

Table S1 Crystal and refinement data for **4c**.

	**4c**
Crystal data	
Chemical formula	$C_{24}H_{16}ClNO_4$
M_r	417.83
Crystal system, space group	Triclinic, *P*
Temperature (K)	150

a, *b*, *c* (Å)	9.7705 (2), 9.9797 (2), 11.2567 (2)
α, β, γ (°)	101.476 (1), 90.020 (1), 108.662 (1)
V (Å^3)	1016.68 (3)
Z	2
Radiation type	Cu *K*α
μ (mm^{-1})	1.93
Crystal size (mm)	0.21 × 0.13 × 0.11
Data collection	
Diffractometer	Bruker D8 VENTURE PHOTON 3 CPAD
Absorption correction	Multi-scan *SADABS* [5]
T_{min}, T_{max}	0.72, 0.82
No. of measured, independent and observed [$I > 2\sigma(I)$] reflections	21831, 4079, 3751
R_{int}	0.031
$(\sin \theta/\lambda)_{max}$ (Å^{-1})	0.625
Refinement	
$R[F^2 > 2\sigma(F^2)]$, $wR(F^2)$, S	0.038, 0.103, 1.09
No. of reflections	4079
No. of parameters	272
H-atom treatment	H-atom parameters constrained
$\Delta\rho_{max}$, $\Delta\rho_{min}$ (e Å^{-3})	0.64, −0.48

Table S2 crystals and refinement data for **4f**.

	**4f**
Crystal data	
Chemical formula	$C_{29}H_{18}ClNO_4$

M_r	479.89
Crystal system, space group	Triclinic, *P*
Temperature (K)	150
a, *b*, *c* (Å)	10.7775 (2), 11.1132 (3), 11.2454 (3)
α, β, γ (°)	109.503 (1), 97.202 (1), 110.116 (1)
V ($Å^3$)	1147.43 (5)
Z	2
Radiation type	Cu *K*α
μ (mm^{-1})	1.79
Crystal size (mm)	0.23 × 0.15 × 0.11
Data collection	
Diffractometer	Bruker D8 VENTURE PHOTON 3 CPAD
Absorption correction	Multi-scan *SADABS* [5]
T_{min}, T_{max}	0.68, 0.82
No. of measured, independent and observed [$I > 2\sigma(I)$] reflections	21047, 4629, 4002
R_{int}	0.041
$(\sin \theta/\lambda)_{max}$ ($Å^{-1}$)	0.627
Refinement	
$R[F^2 > 2\sigma(F^2)]$, $wR(F^2)$, S	0.050, 0.142, 1.11
No. of reflections	4629
No. of parameters	316
H-atom treatment	H-atom parameters constrained
$\Delta\rho_{max}$, $\Delta\rho_{min}$ (e $Å^{-3}$)	0.49, −0.29

Computer programs: *APEX4* [4], *SAINT* [4], *SHELXT* [6], *SHELXL-2018/1* [7], *DIAMOND* [8], *SHELXTL* [4].

4. *Disk diffusion method*

The antibacterial activities of the synthesized isoxazolic compounds against *Staphylococcus aureus* (CECT 976), *Bacillus subtilis* (DSM 6633), and *Escherichia coli* (K12), were assessed via the conventional agar disk diffusion technique, according to protocols published in the literature with minor modifications [9–11]. Stock solutions of the synthesized isoxazoles were prepared in 1 mL of anhydrous DMF to obtain the necessary concentration (10 mg/mL). Then, 10 µL of the compounds were added to the Whatman Filter Paper disk (6 mm diameter) and dried for 10 min. Ampicillin and streptomycin were used as standard antibacterial agents. DMF alone was used as a control, and it showed no effect on the growth of microorganisms. After that, the plates were incubated for 24 hours at 37 °C for the bacteria. Lastly, a transparent ruler was used to measure the zone of inhibition surrounding the disk (in mm). The results are represented by the average diameter from two experiments.

5. *Characterization data*

5.1 5-(2-hydroxy*benzoyl)*-4-phenyl-3-(4-methoxyphenyl) isoxazole (3b):

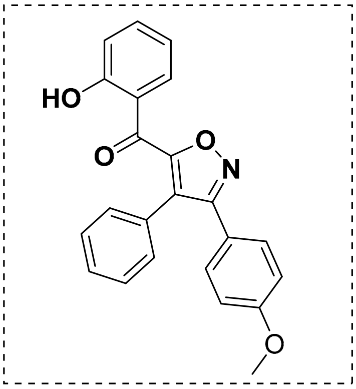

Yellow solid; **Yield** (59 %); **M.p**: 117-115 °C; **^{1}H NMR (300 MHz, $CDCl_3$)** (δ/ppm): **^{1}H NMR (300 MHz, $CDCl_3$)** (δ in ppm): 3.93 (s, 3H, OCH_3), 6.95 (td, 1H, H_{ar}, ^{4}J = 1.2 Hz, ^{3}J = 7.5 Hz), 7.05 (dd, 1H, H_{ar}, ^{4}J = 0.9 Hz, ^{3}J = 8.4 Hz), 7.26 (d, 1H, H_{ar}, J = 3 Hz), 7.28-7.33 (m, 3H, H_{ar}), 7.37-7.44 (m, 4H, H_{ar}), 7.53-7.59 (m, 2H, H_{ar}), 8.01 (dd, 1H, H_{ar}, ^{4}J = 1.5 Hz, ^{3}J = 4.2 Hz), 11.63 (s, 1H, OH).

5.2 *Spectral analysis of isoxazole-ester (4a-c)*

5-(2-acetoxybenzoyl)-4-phenyl-3-(p-tolyl) isoxazole (4a):

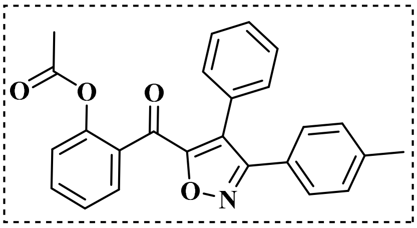

White solid; **Yield** (95 %); **m.p**: 142-144 °C; **IR-FT (neat, *v*, cm^{-1})**: 1449 (C=C$_{Ar}$), 1179 (C-O), 1667 (C=O$_{(ketone)}$), 1753 (C=O$_{(ester)}$), 1603 (C=N); **^{1}H NMR (300 MHz, CDCl$_3$) (*δ*/ppm)**: 2.20 (s, 3H, CH$_{3(ester)}$), 3.38 (s, 3H, CH$_3$), 7.15-7.20 (m, 3H, H$_{Ar}$), 7.52-7.57 (td, 1H, H$_{Ar}$, ^4J= 1.5 Hz, ^3J= 8.1 Hz), 7.63-7.66 (dd, 1H, H$_{Ar}$, ^4J= 1.5 Hz, ^3J= 7.8 Hz); **^{13}C NMR (75 MHz, CDCl$_3$) (*δ*/ppm)**: 20.80 (**C**H$_3$), 21.40 (**C**H$_{3(ester)}$), 123.26, 123.38, 124.92, 125.68, 128.11, 128.40, 128.53, 128.61, 128.67, 129.38, 129.96, 130.17, 130.96, 133.83, 140.12, 149.37, 161.78 (**C**=N), 162.30, 168.91 (**C**=O$_{(ester)}$), 181.78 (**C**=O$_{(ketone)}$); **MS (ESI)** (*m/z*): mass calculated for [C$_{25}$H$_{19}$NO$_4$+H]$^+$: 398.44; found: 398.39.

5-(2-acetoxybenzoyl)-3-(4-methoxyphenyl)-4-phenyl isoxazole (4b):

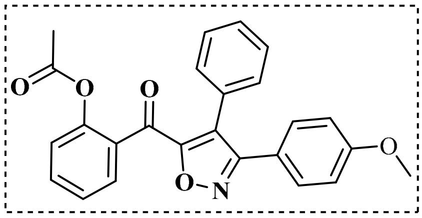

White solid; **Yield** (90 %); **m.p** : 140-142 °C; **IR-FT (neat, *v*, cm^{-1}):** 1185 (C-O), 1452 (C=C$_{Ar}$), 1666 (C=O$_{(ketone)}$), 1756(C=O$_{(ester)}$), 1604 (C=N), 2918 (C-H of CH$_3$), 3051 (C-H$_{Ar}$); **^{1}H NMR (300 MHz, CDCl$_3$) (*δ*/ppm)**: 2.20 (s, 3H, CH$_{3(ester)}$), 3.83 (s, 3H, OCH$_3$), 7.17-7.41 (m, 11H, H$_{Ar}$), 7.51-7.57 (m, 1H, H$_{Ar}$), 7.64 (dd, 1H, H$_{Ar}$, ^4J= 1.5 Hz, ^3J= 7.8 Hz); **^{13}C NMR (75 MHz, CDCl$_3$) (*δ*/ppm)**: 20.80 (**C**H$_{3(ester)}$), 55.29 (O**C**H$_3$), 114.13, 120.12, 123.13, 125.67, 128.20, 128.62, 129.41, 130.03, 130.11, 130.18, 130.95, 133.81, 149.36, 160.92 (**C**=N), 161.75, 161.94, 168.92 (**C**=O$_{(ester)}$), 181.79 (**C**=O$_{(ketone)}$); **MS (ESI)** (*m/z*): mass calculated for [C$_{25}$H$_{19}$NO$_5$+H]$^+$: 414.44; found: 414.36.

5-(2-acetoxybenzoyl)- 3-(4-chlorophenyl)-4-phenyl isoxazole (4c):

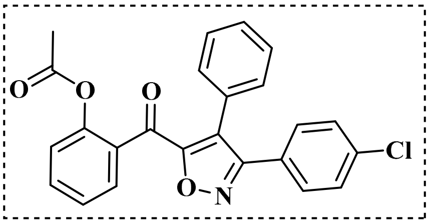

White solid; **Yield** (85 %); **m.p**: 128-130°C; **IR-FT (neat, *ν*, cm^{-1})**: 1188 (C-O), 1450 (C=C$_{Ar}$), 1666 (C=O$_{(ketone)}$), 1764 (C=O$_{(ester)}$), 1609 (C=N), 2914 (C-H of CH$_3$), 3077 (C-H$_{Ar}$); **^{1}H NMR (300 MHz, CDCl$_3$) (*δ*/ppm)**: 2.21 (s, 3H, CH$_{3(ester)}$), 7.17-7.40 (m, 11H, H$_{Ar}$), 7.64 (td, 1H, H$_{Ar}$, ^4J= 1.8 Hz, ^3J= 7.5Hz), 7.63 (dd, 1H, H$_{Ar}$, *J*= 1.8 Hz, *J*= 7.8Hz); **^{13}C NMR (75 MHz, CDCl$_3$) (*δ*/ppm)**: 20.83 (<u>C</u>H$_3$), 123.42, 125.70, 126.32, 127.67, 128.58, 128.87, 129.03, 129.21, 129.93, 130.09, 130.94, 136.28, 149.37, 161.39 (<u>C</u>=N), 162.01, 168.95 (<u>C</u>=O$_{(ester)}$), 181.55 (<u>C</u>=O$_{(ketone)}$); **MS (ESI)** (*m/z*): mass calculated for [C$_{24}$H$_{16}$ClNO$_4$+H]$^+$: 418.85, found: 417.34.

5.3 ***Spectral analysis of isoxazole-ester 4(d-f)***

***5-(2-benzoyloxybenzoyl)*-3-(p-tolyl*)-4-phenyl isoxazole* (4d):**

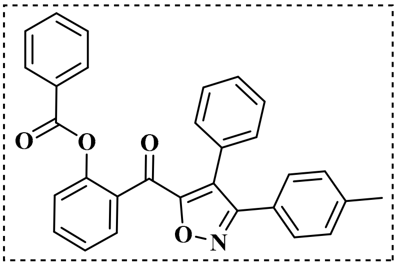

White solid; **Yield (92 %)**; **m.p**: 118-120°C; **IR-FT (neat, *ν*, cm^{-1})**: 1188 (C-O), 1449 (C=C$_{Ar}$), 1667 (C=O$_{(ketone)}$), 1754 (C=O$_{(ester)}$), 1600 (C=N), 1609 (C=N), 2928 (C-H of CH$_3$), 3037 (C-H$_{Ar}$); **^{1}H NMR (300 MHz, CDCl$_3$) (*δ*/ppm)**: 2.23 (s, 3H, CH$_3$), 7.04-7.15 (m, 7H, H$_{Ar}$), 7.23-7.45 (m, 7H, H$_{Ar}$), 7.54-7.66 (m, 2H, H$_{Ar}$), 7.75 (dt, 1H, H$_{Ar}$, ^4J= 1.5 Hz, ^3J= 7.8 Hz), 8.03 (dt, 1H, H$_{Ar}$, ^4J= 1.5 Hz, ^3J= 8.4 Hz); **^{13}C NMR (75 MHz, CDCl$_3$) (*δ*/ppm)**: 21.38 (<u>C</u>H$_3$), 122.90, 123.35, 124.80, 126.02, 127.81, 128.25, 128.50, 128.55, 128.67, 129.16, 130.14, 130.15, 130.40, 130.75, 133.83, 133.89, 139.90, 149.48, 161.89 (<u>C</u>=N), 162.36, 164.36 (<u>C</u>=O$_{(ester)}$), 182.07 (<u>C</u>=O$_{(ketone)}$); **MS (ESI)** (*m/z*): mass calculated for [C$_{30}$H$_{21}$NO$_4$+H]$^+$: 460.51, found: 461.39.

***5-(2-benzoyloxybenzoyl)*-3-(4-méthoxyphenyl*)-4-phenyl isoxazole* (4e):**

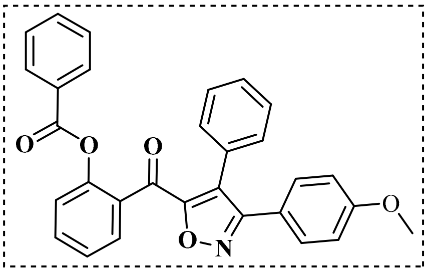

White solid; **Yield** (89 %); **m.p**: 124-126 °C; **IR-FT (neat, *ν*, cm^{-1})**: 1195 (C-O), 1453 (C=C_{Ar}), 1670 (C=$O_{(ketone)}$), 1741 (C=$O_{(ester)}$), 1597 (C=N), 2938 (C-H of CH_3), 3065 (C-H_{Ar}); **^{1}H NMR (300 MHz, $CDCl_3$) (*δ*/ppm)**: 3.9 (s, 3H, OCH_3), 6.79 (dd, 1H, H_{Ar}, ^{4}J= 3.6 Hz, ^{3}J= 8.7 Hz), 6.97 (dd, 1H, H_{Ar}, ^{4}J= 2.1 Hz, ^{3}J= 10.8 Hz), 7.26-7.46 (m, 10H, H_{Ar}), 7.54-7.78 (m, 4H, H_{Ar}), 8.03 (d, 2H, H_{Ar}, *J*= 7.2 Hz); **^{13}C NMR (75 MHz, $CDCl_3$) (*δ*/ppm)** : 55.25 (O$\underline{C}$H3), 111.60, 113.90, 120.01, 120.01, 120.82, 122.74, 123.36, 123.42, 126.02, 126.10, 127.42, 127.89, 128.19, 128.28, 128.39, 128.53, 128.56, 128.58, 128.68, 128.79, 130.00, 130.10, 130.14, 130.37, 130.42, 130.74, 133.82, 133.88, 133.95, 134.07, 149.48, 156.14, 160.96 ($\underline{C}$-O-CH_3), 161.99 ($\underline{C}$=N), 164.35 ($\underline{C}$=$O_{(ester)}$), 182.08 ($\underline{C}$=$O_{(ketone)}$); **MS (ESI)** (*m/z*): mass calculated for $[C_{30}H_{21}NO_5+H]^+$: 476.51, found: 476.43.

5-(2-benzoyloxybenzoyl)-*3-(4-chlorophenyl*)-4-phenyl isoxazole **(4f):**

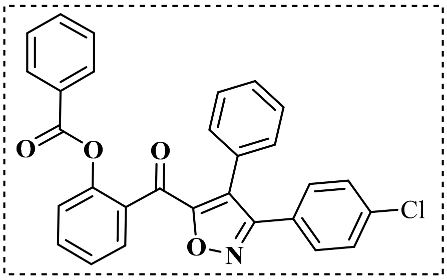

White solid; Yield (91 %); **m.p**: 126-130 °C; **IR-FT (neat, *ν*, cm^{-1})**: 1182 (C-O), 1452 (C=C_{Ar}), 1677 (C=$O_{(ketone)}$), 1760 (C=$O_{(ester)}$), 1609 (C=N), 3067 (C-H_{Ar}); **^{1}H NMR (300 MHz, $CDCl_3$) (*δ*/ppm)**: 7.10 (dt, 2H, H_{Ar}, ^{4}J= 2.1 Hz, ^{3}J= 8.7 Hz), 7.14 (dt, 2H, H_{Ar}, ^{4}J= 2.1 Hz, ^{3}J= 6.6 Hz), 7.24-7.45 (m, 9H, H_{Ar}), 7.59-7.67 (m, 1H, H_{Ar}), 7.75-7.80 (m, 1H, H_{Ar}), 7.77 (dd, 1H, H_{Ar}, *J*= 1.5 Hz*, J*= 7.8 Hz), 8.01-8.08 (m, 1H, H_{Ar}); **^{13}C NMR (75 MHz, $CDCl_3$) (*δ*/ppm)** : 122.69, 123.41, 126.13, 126.21, 127.34, 128.46, 128.57, 128.80, 128.83, 129.90, 130.07, 130.15, 130.25, 130.78, 133.93, 134.13, 136.08, 149.52, 161.39 ($\underline{C}$=N), 162.22, 164.30 ($\underline{C}$=$O_{(ester)}$), 181.88 ($\underline{C}$=$O_{(ketone)}$); **MS (ESI)** (*m/z*): mass calculated for $[C_{29}H_{18}ClNO_4+H]^+$: 480.92, found: 481.36.

6. *Copies of* [1]*H,* [13]*C NMR and MS spectra of all new compounds*

5-(2-hydroxy*benzoyl)*-4-phenyl-3-(4-methoxyphenyl)isoxazole (3b):

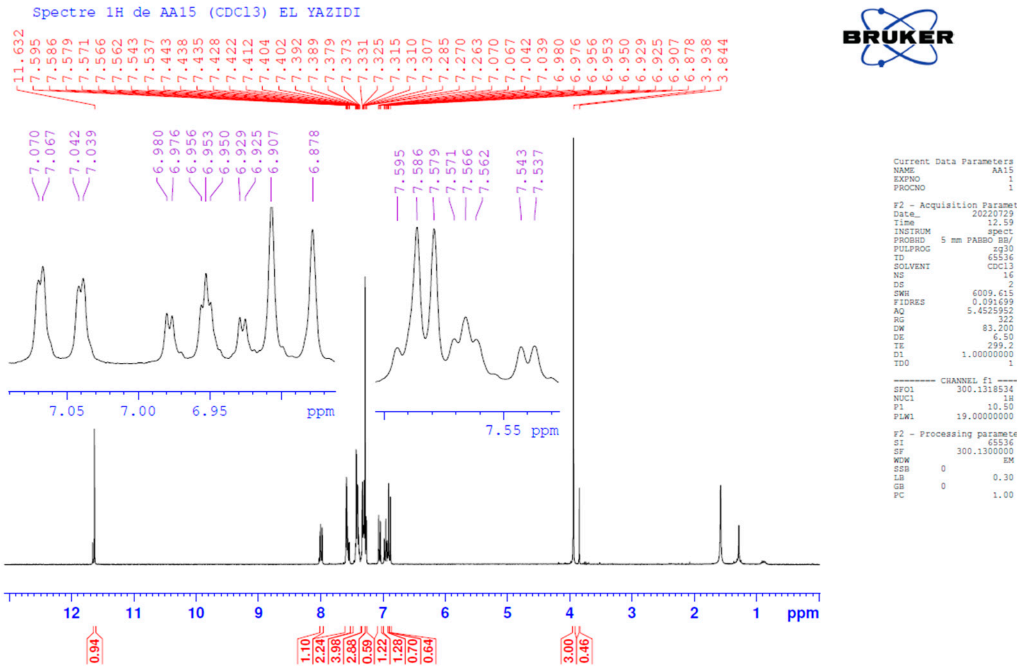

Figure S1. ^{1}H NMR spectrum (300 MHz, $CDCl_3$) of compound (**3b**)

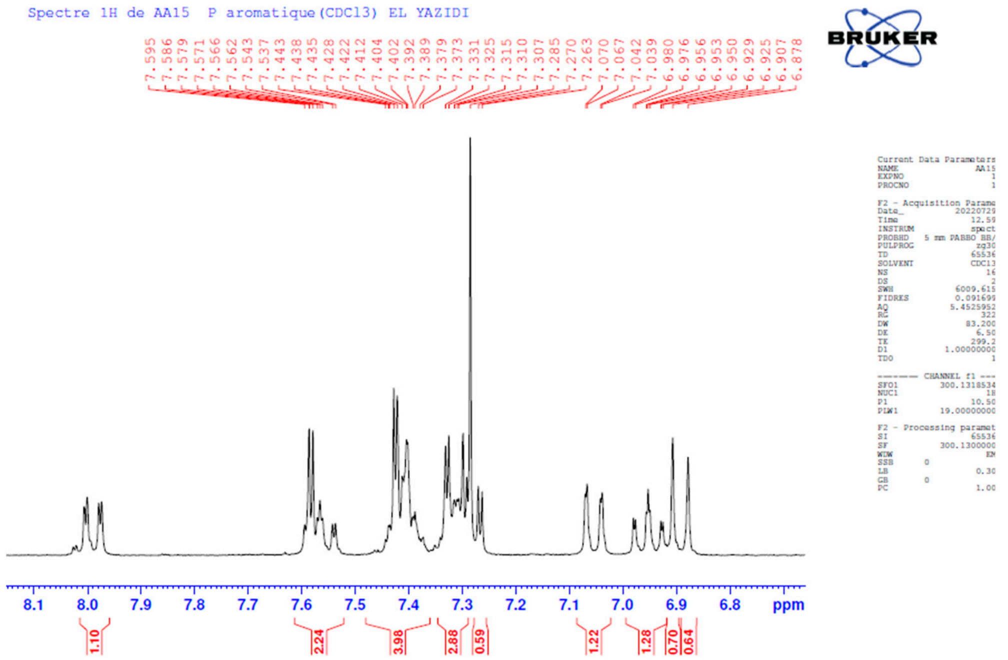

Figure S2. Aromatic enlarged region of ^{1}H NMR spectrum of compound (**3b**)

5-(2-acetoxybenzoyl)-4-phenyl-3-(p-tolyl)-isoxazole (4a):

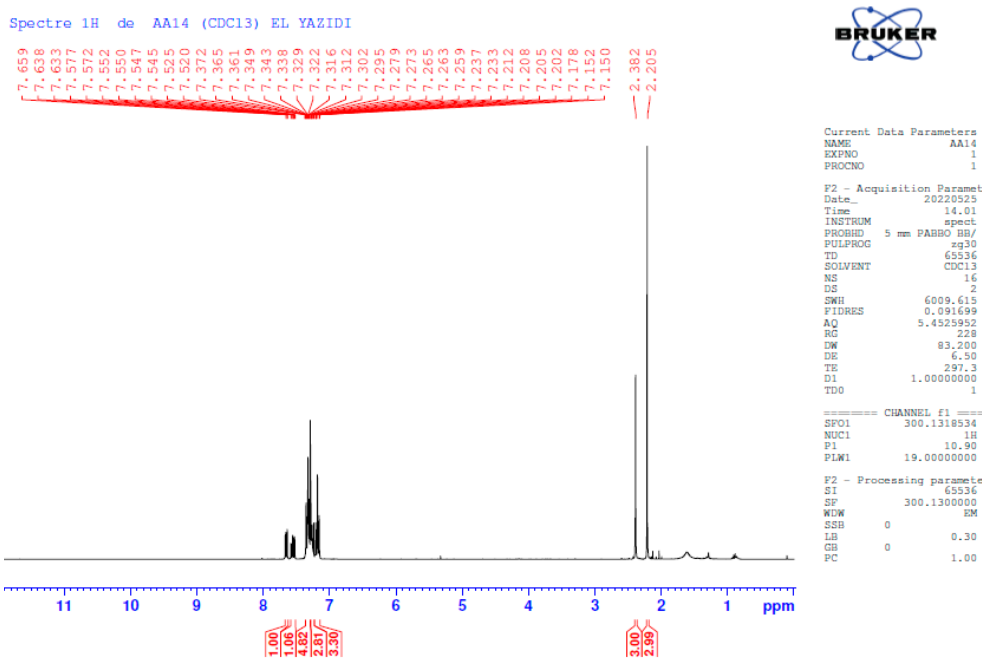

Figure S3. ^{1}H NMR spectrum (300 MHz, $CDCl_3$) of compound (**4a**)

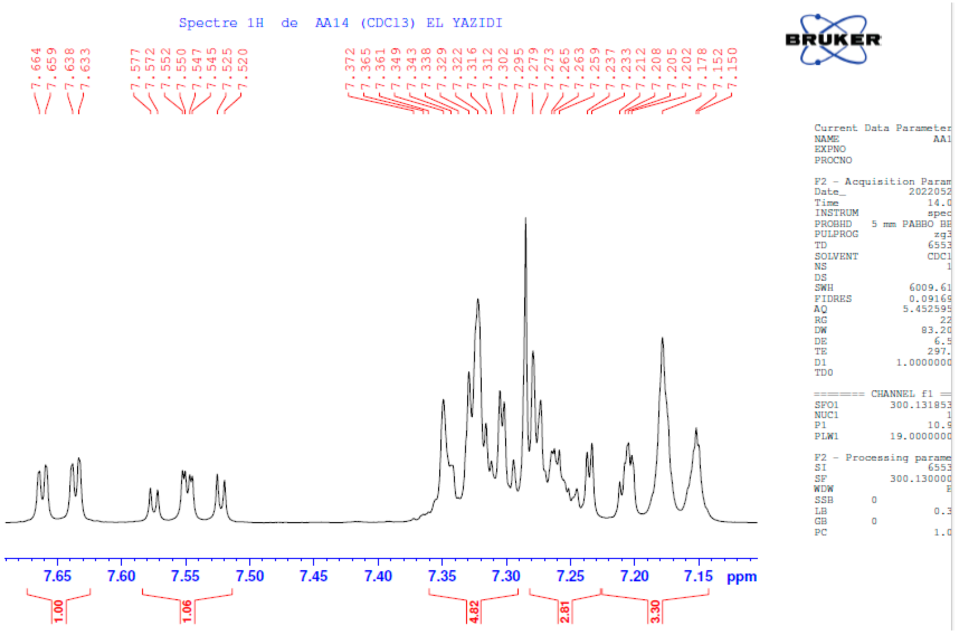

Figure S4. Aromatic enlarged region of ^{1}H NMR spectrum of compound (**4a**)

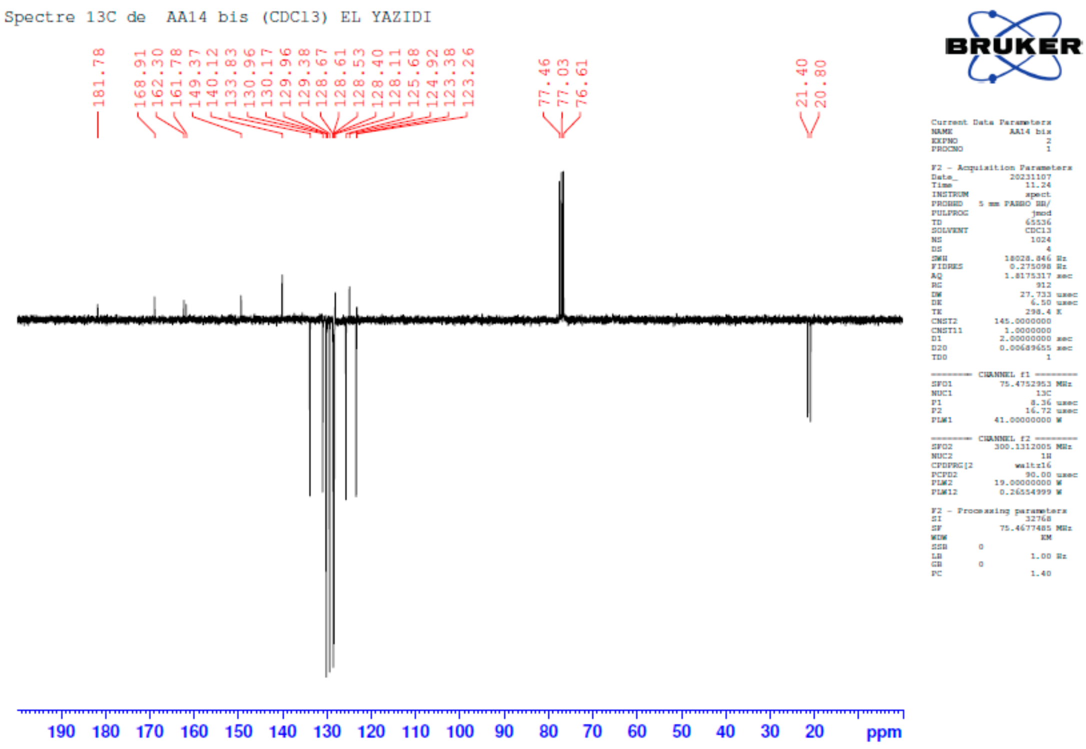

Figure S5. ^{13}C NMR spectrum (75 MHz, $CDCl_3$) of compound (**4a**)

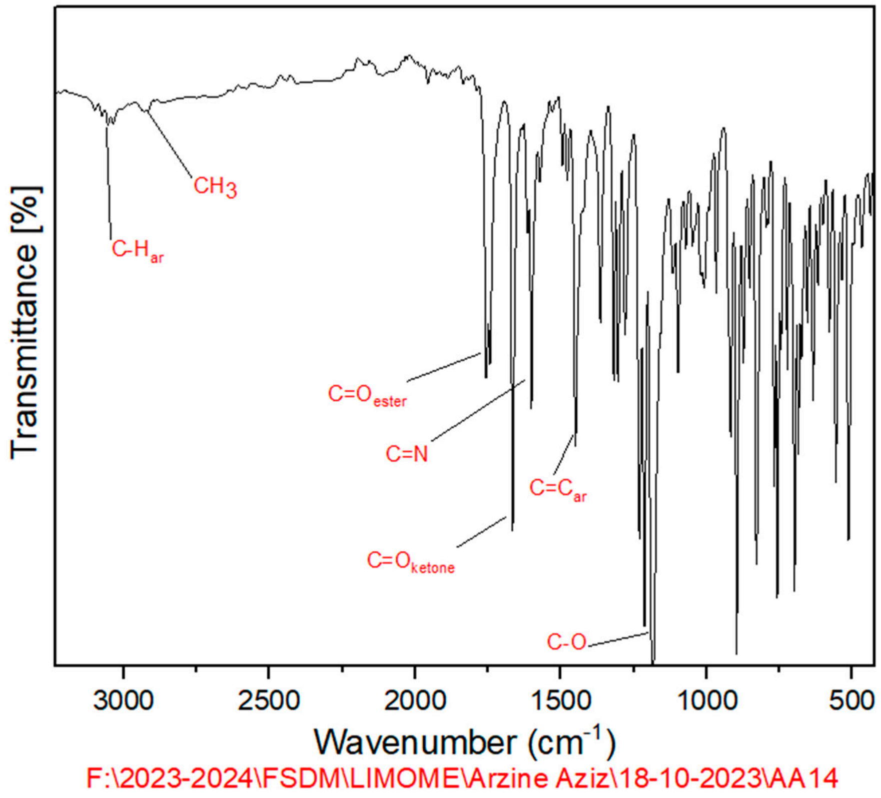

Figure S6. IF spectrum of compound (**4a**)

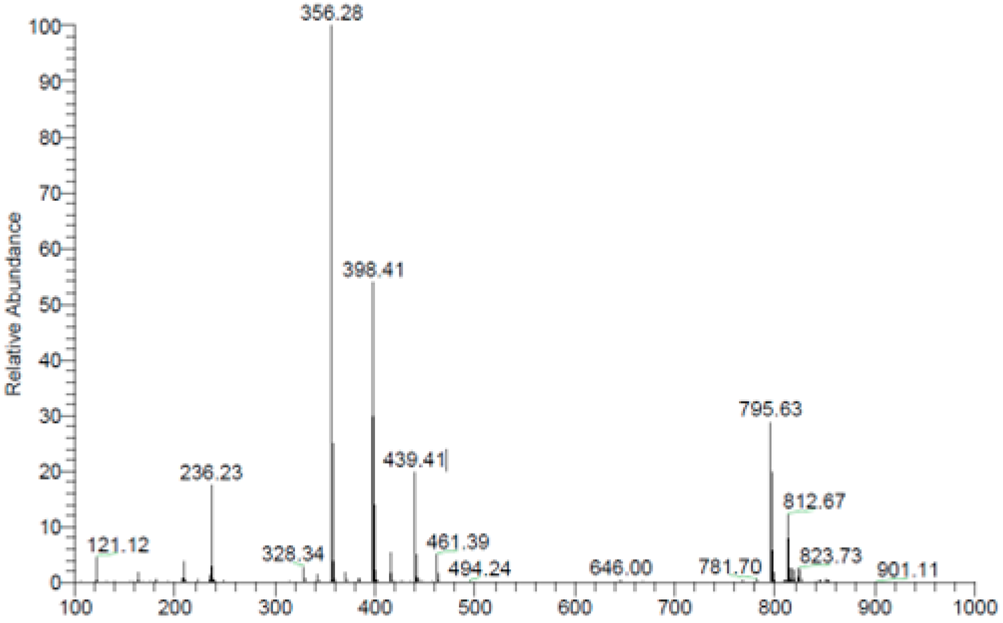

Figure S7. Mass spectrum of compound (**4a**)

5-(2-acetoxybenzoyl)-3-(4-methoxyphenyl)-4-phenyl isoxazole (4b):

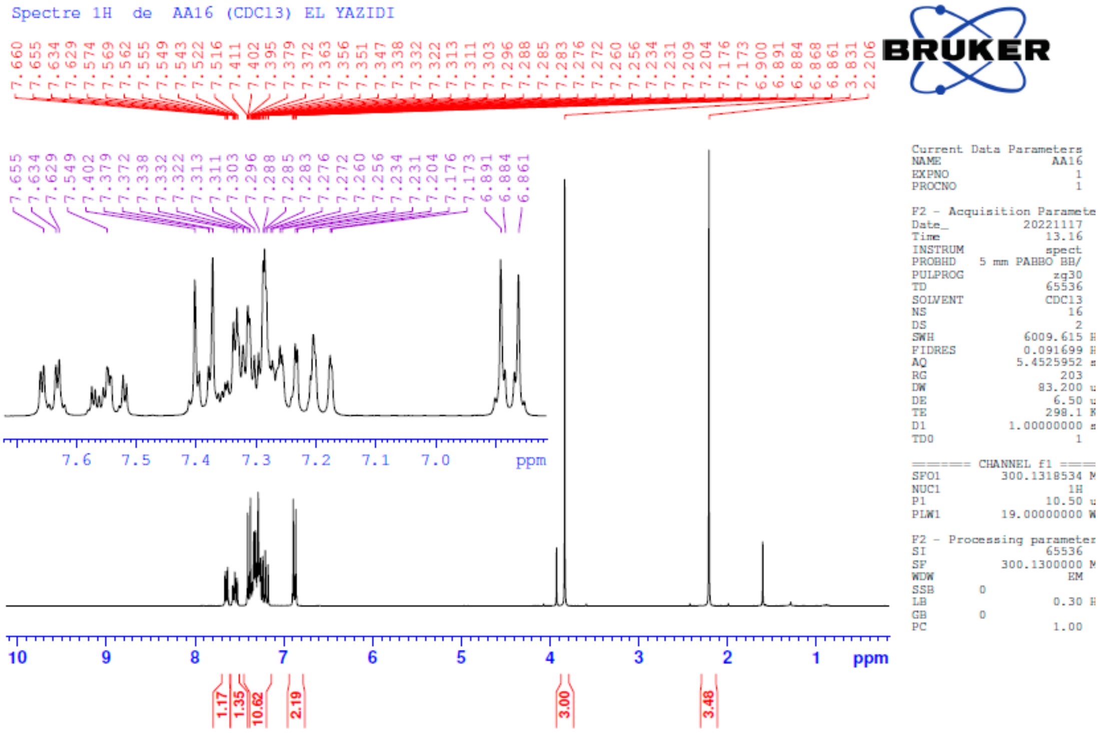

Figure S8. ^{1}H NMR spectrum (300 MHz, $CDCl_3$) of compound (**4b**)

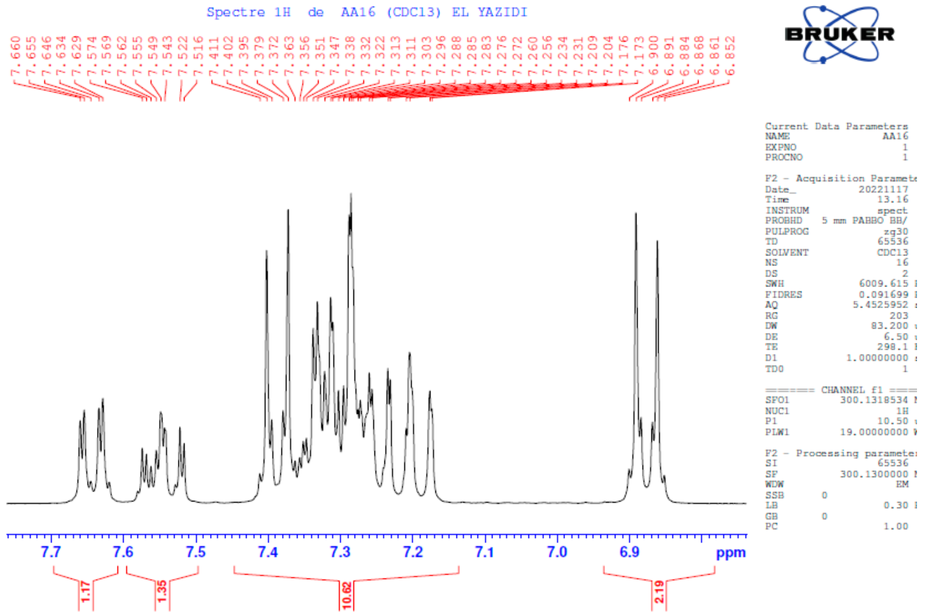

Figure S9. Aromatic enlarged region of ^{1}H NMR spectrum of compound (**4b**)

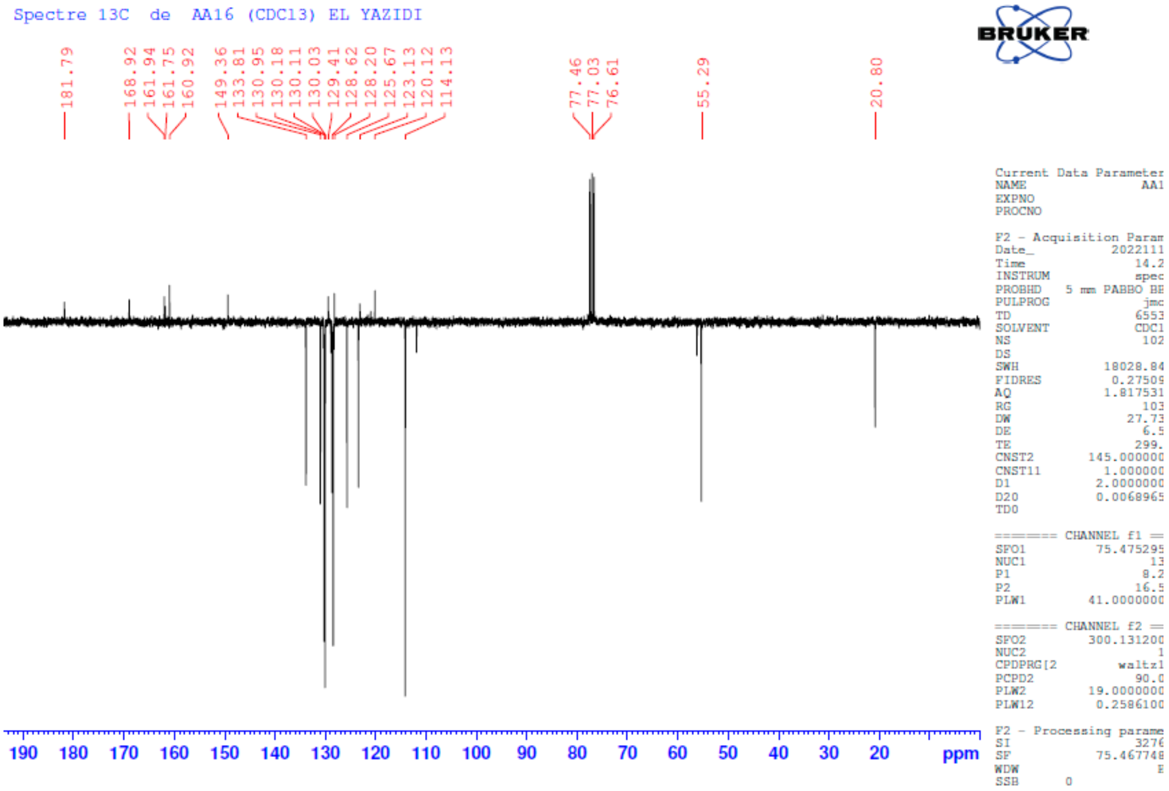

Figure 10. ^{13}C NMR spectrum (75 MHz, $CDCl_3$) of compound (**4b)**

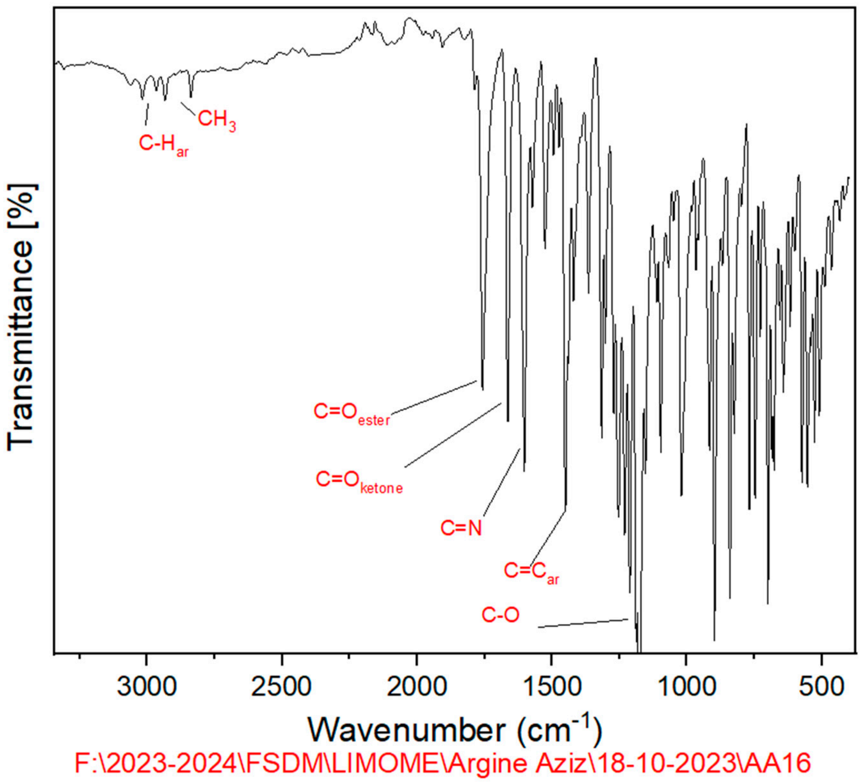

Figure S11. IF spectrum of compound (**4b)**

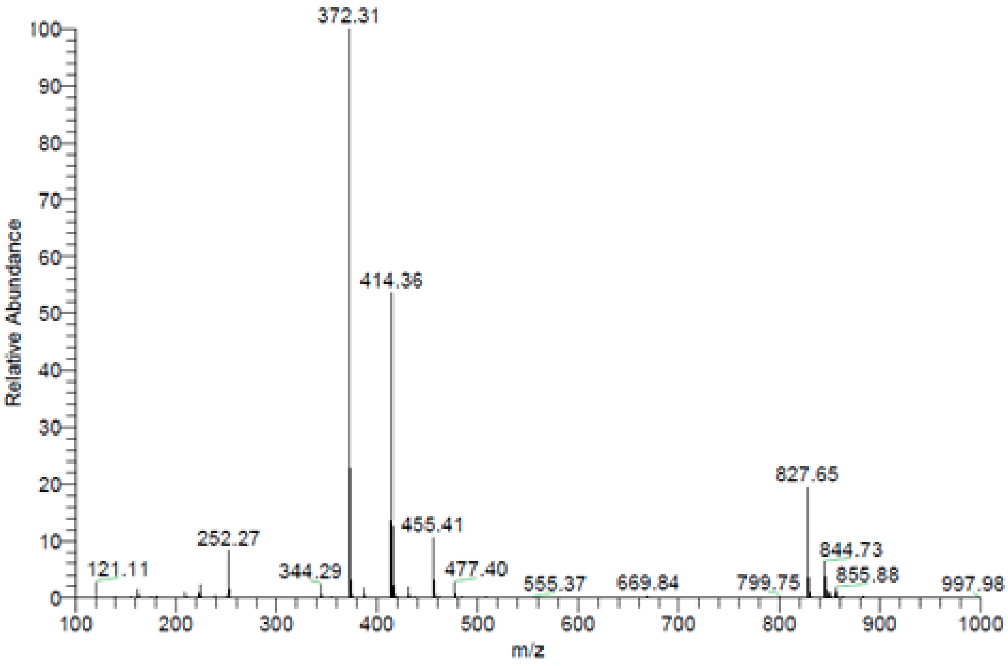

Figure S12. Mass spectrum of compound (**4b**)

5-(2-acetoxybenzoyl)- 3-(4-chlorophenyl)-4-phenyl isoxazole (4c):

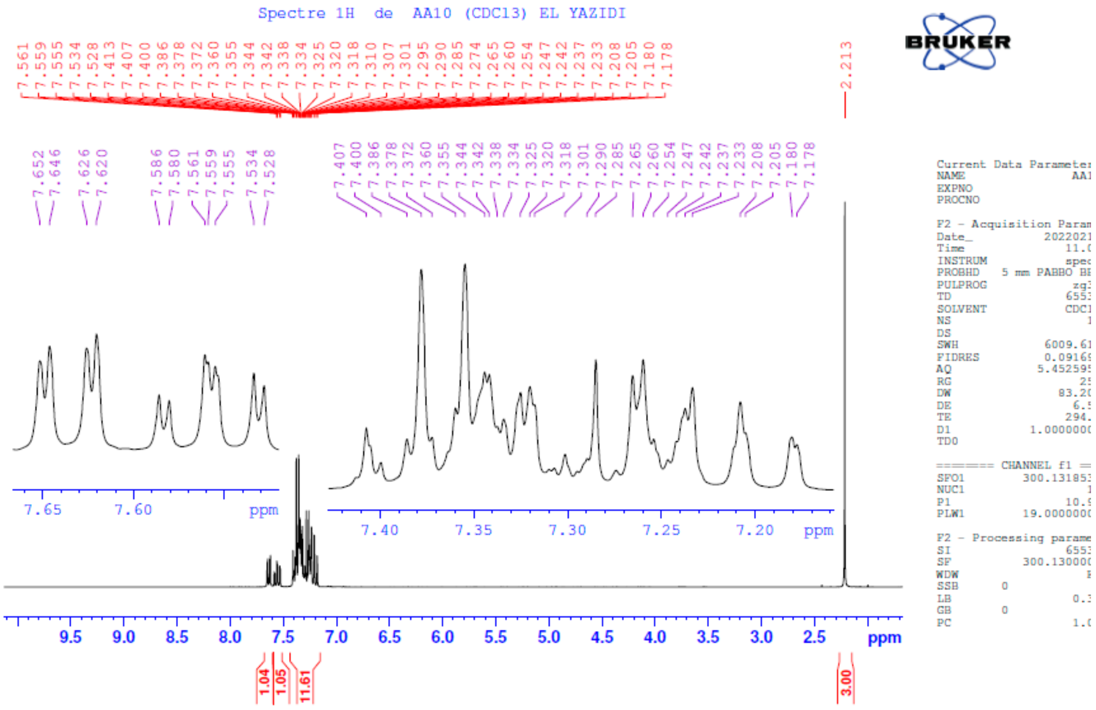

Figure S13. ^{1}H NMR spectrum (300 MHz, $CDCl_3$) of compound (**4c**)

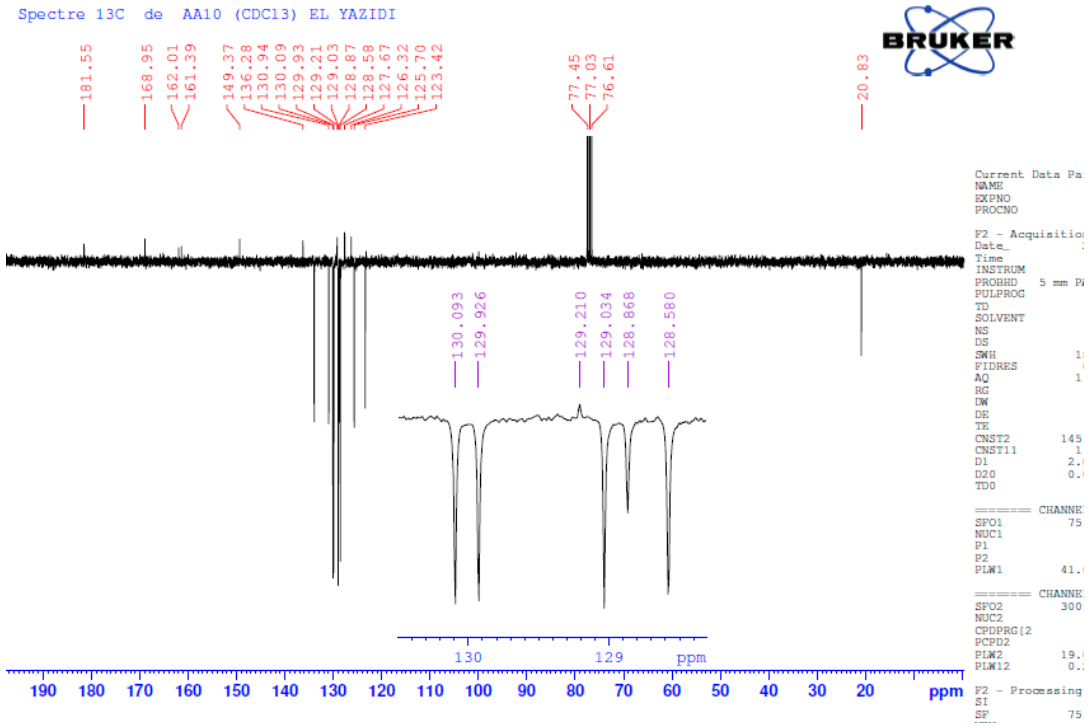

Figure S14. ^{13}C NMR spectrum (75 MHz, $CDCl_3$) of compound (**4c**)

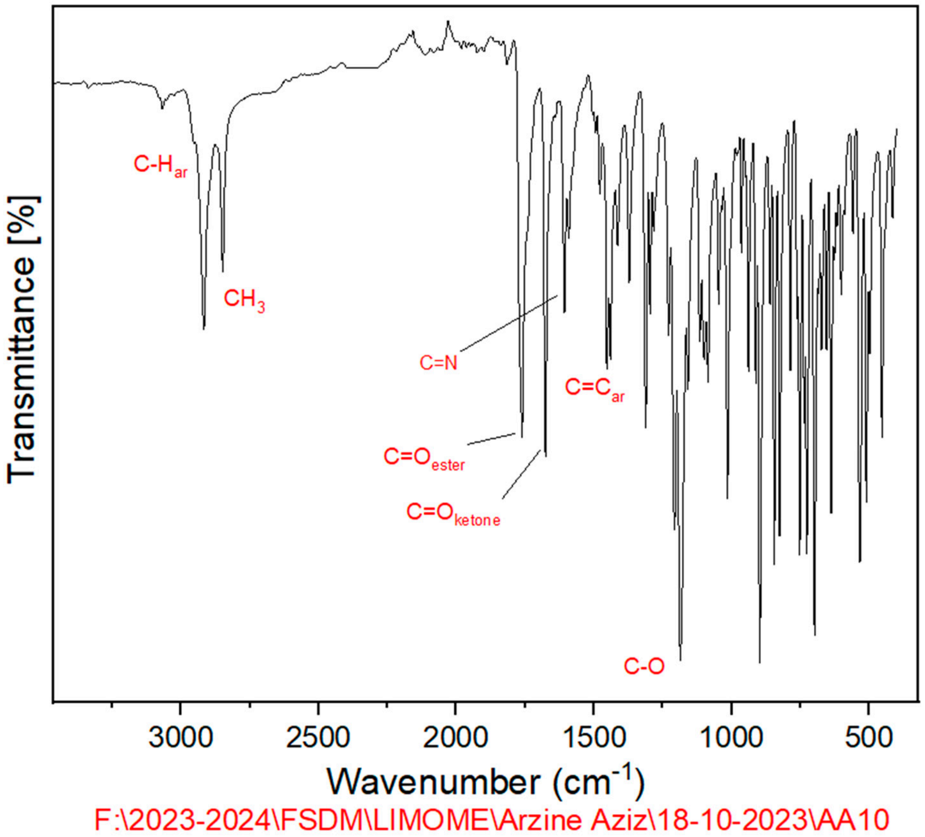

Figure S15. IF spectrum of compound **(4c)**

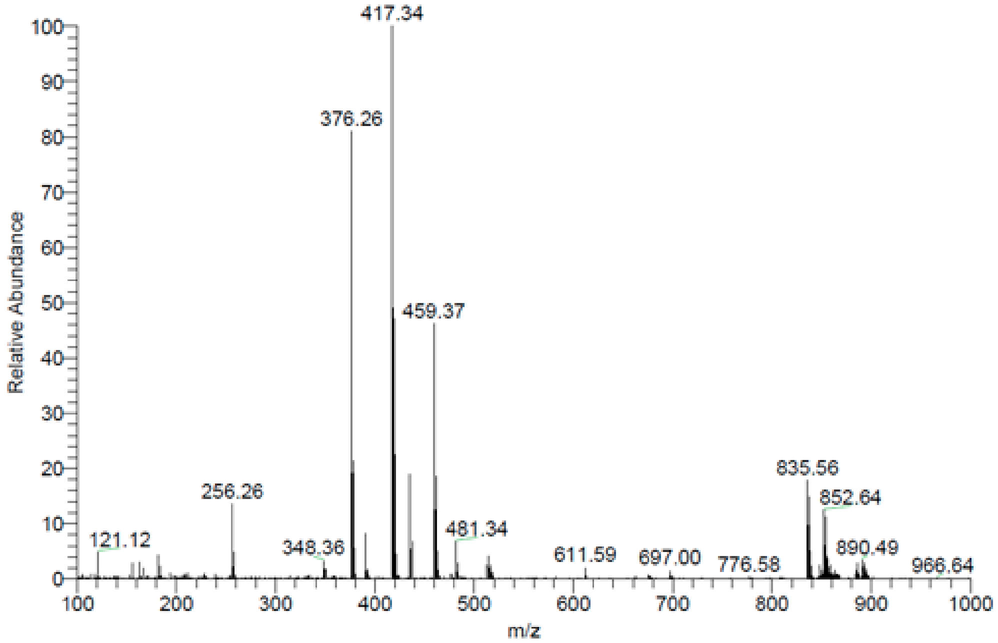

Figure S16. Mass spectrum of compound (**4c**)

***5-(2-benzoyloxybenzoyl)*-3-(p-tolyl)*-4-phenyl isoxazole* (4d):**

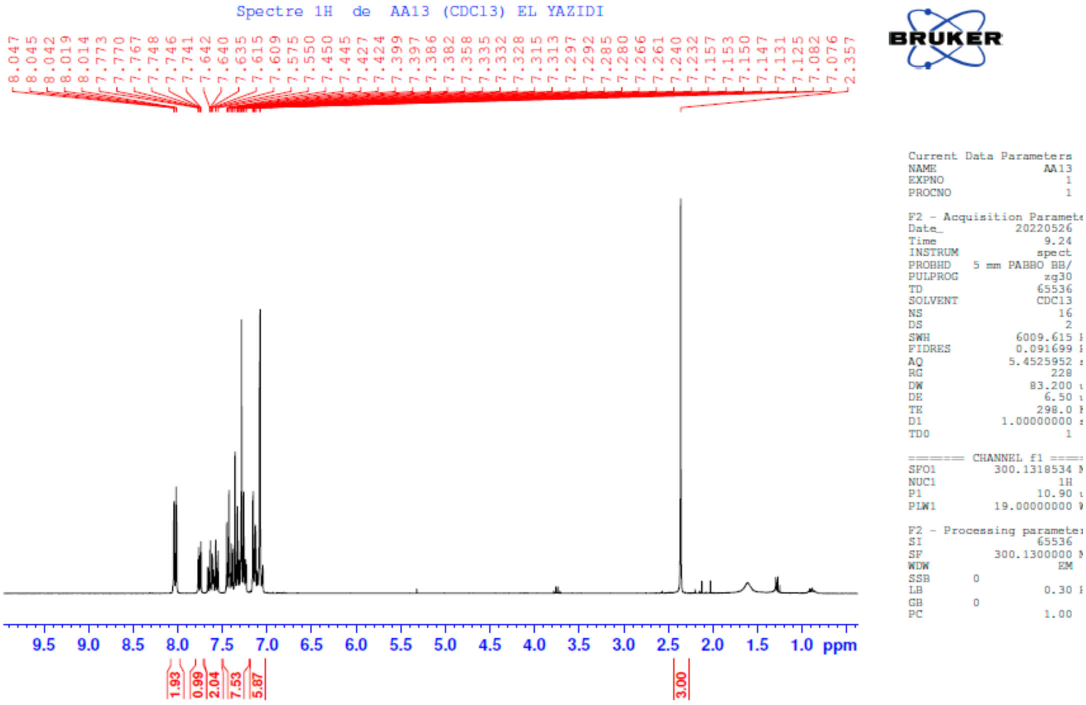

Figure S17. ¹H NMR spectrum (300 MHz, $CDCl_3$) of compound (**4d**)

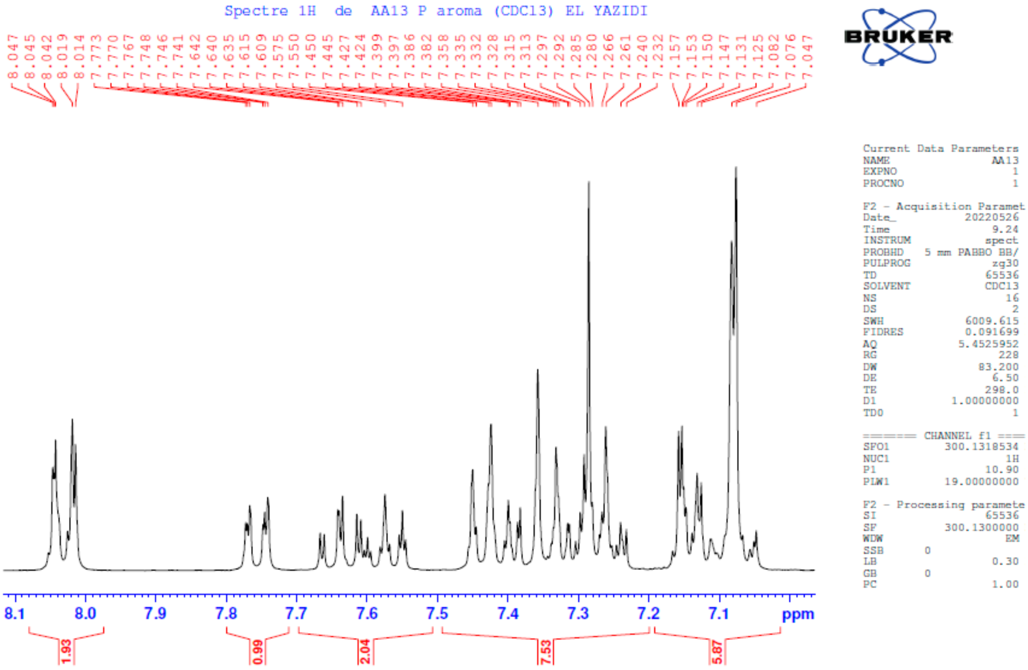

Figure S18. Aromatic enlarged region of ¹H NMR spectrum of compound (**4d**)

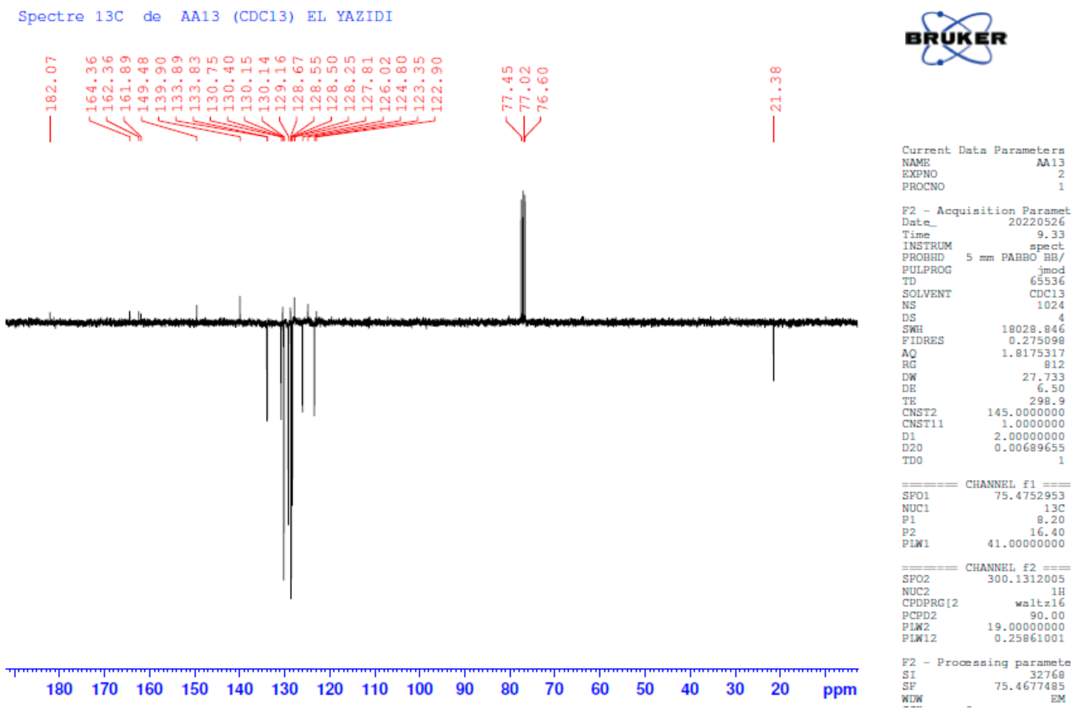

Figure S19. ^{13}C NMR spectrum (75 MHz, $CDCl_3$) of compound (**4d**)

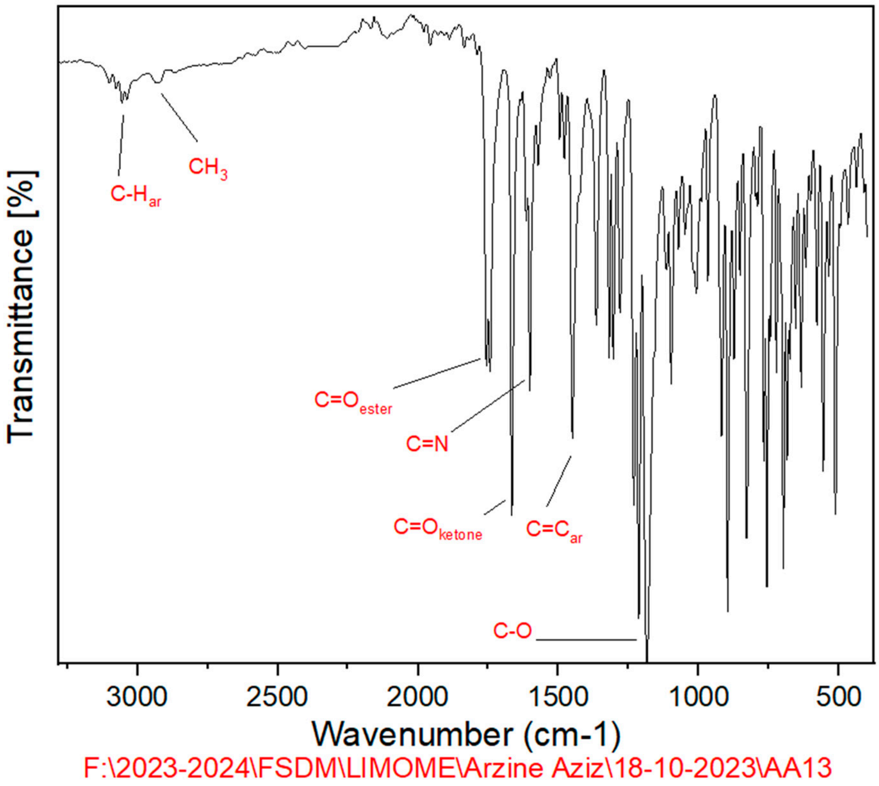

Figure S20. IF spectrum of compound (**4d**)

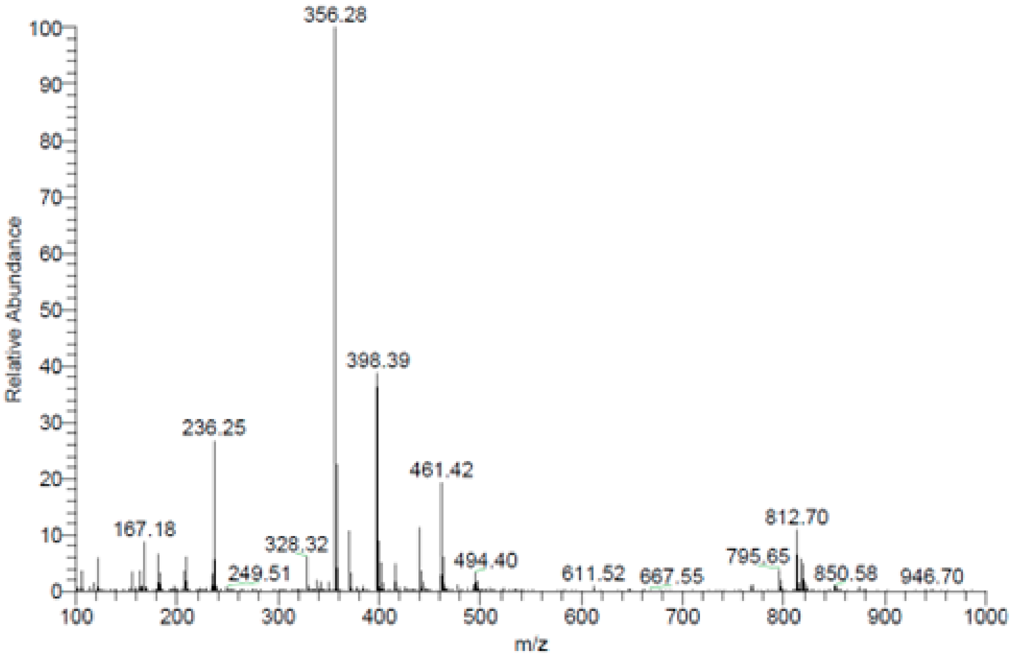

Figure S21. Mass spectrum of compound (**4d**)

***5-(2-benzoyloxybenzoyl)*-3-(4-méthoxyphenyl*)-4-phenyl isoxazole* (4e):**

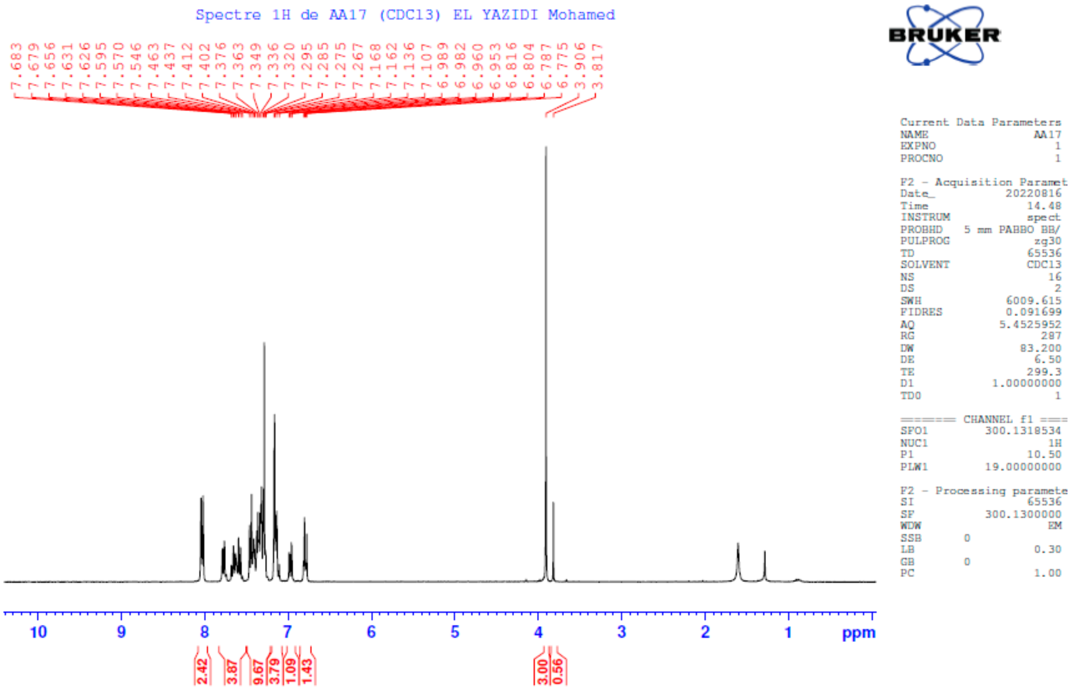

Figure S22. ^{1}H NMR spectrum (300 MHz, $CDCl_3$) of compound (**4e**)

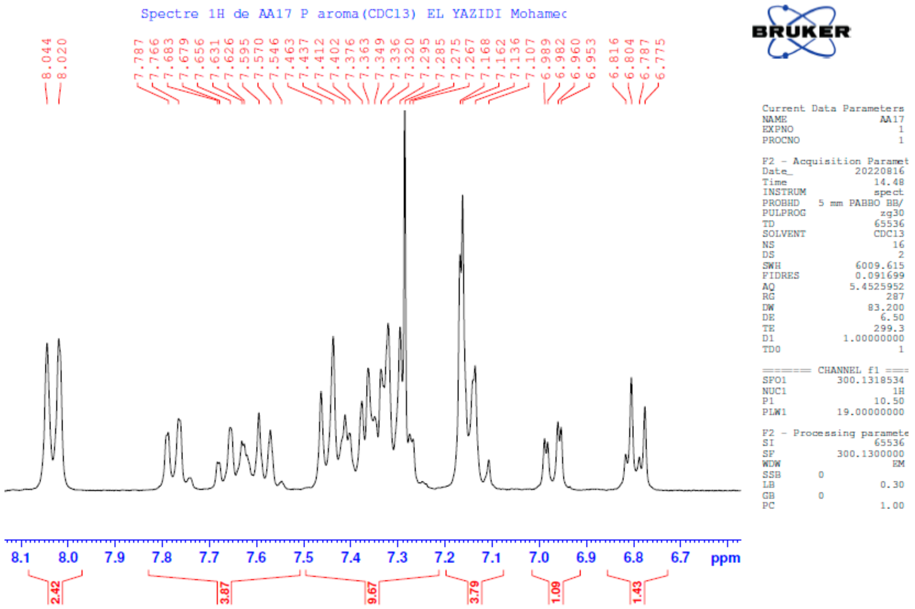

Figure S23. Aromatic enlarged region of ^{1}H NMR spectrum of compound (**4e**)

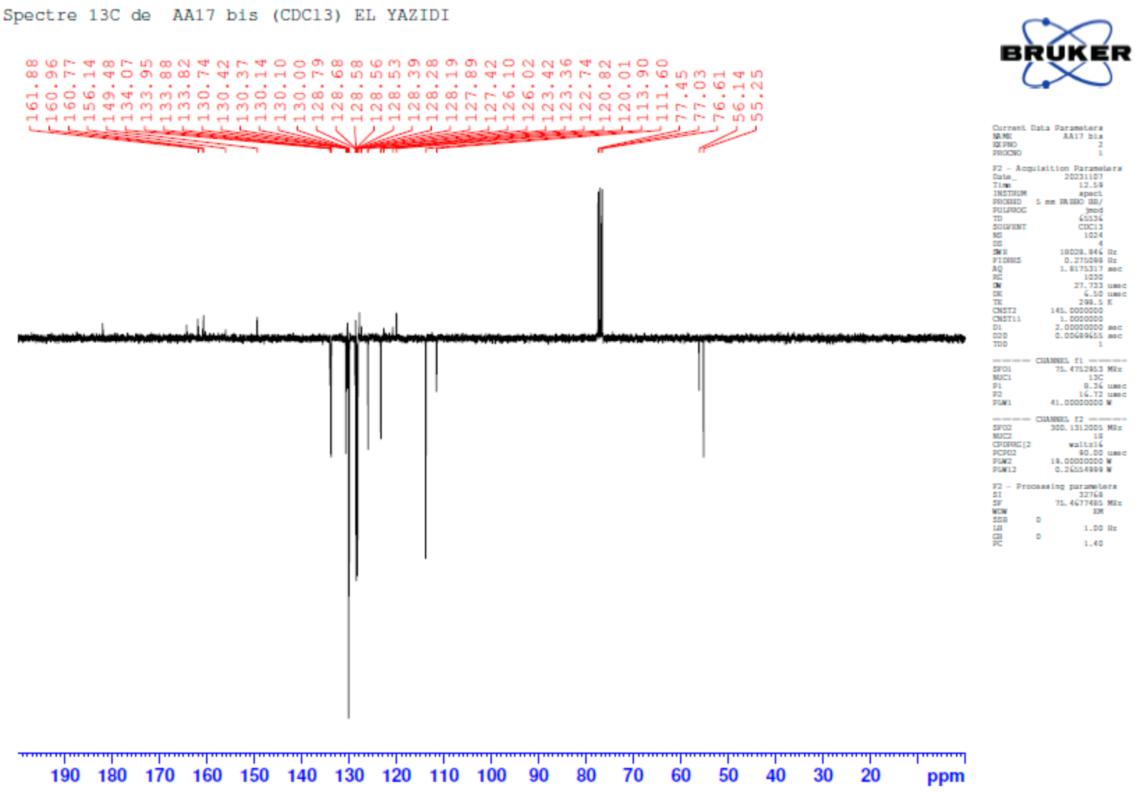

Figure S24. ^{13}C NMR spectrum (75 MHz, $CDCl_3$) of compound (**4e**)

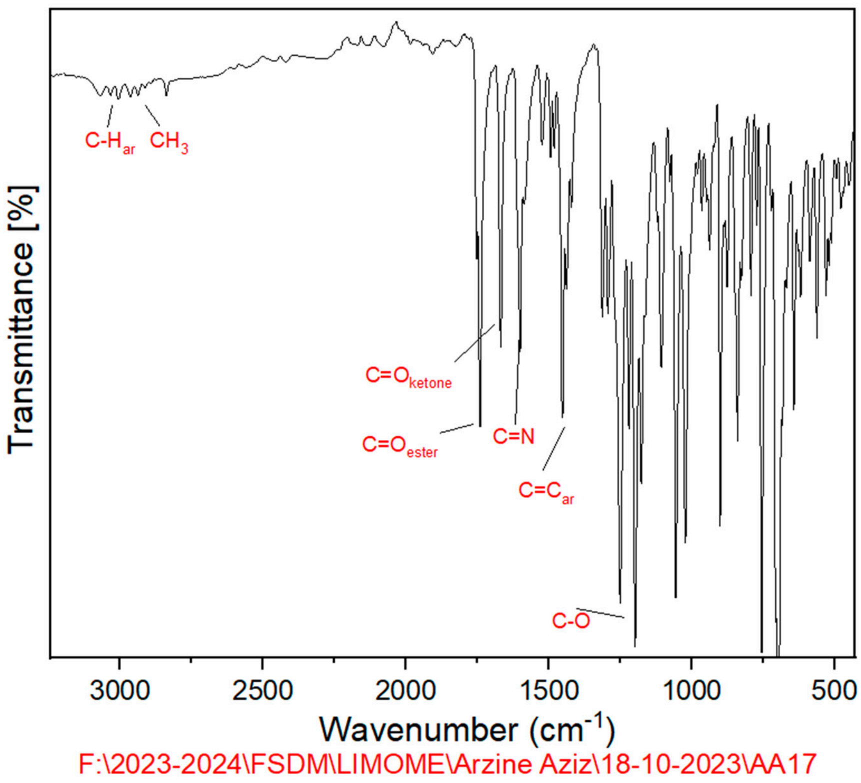

Figure 25. IF spectrum of compound **(4e)**

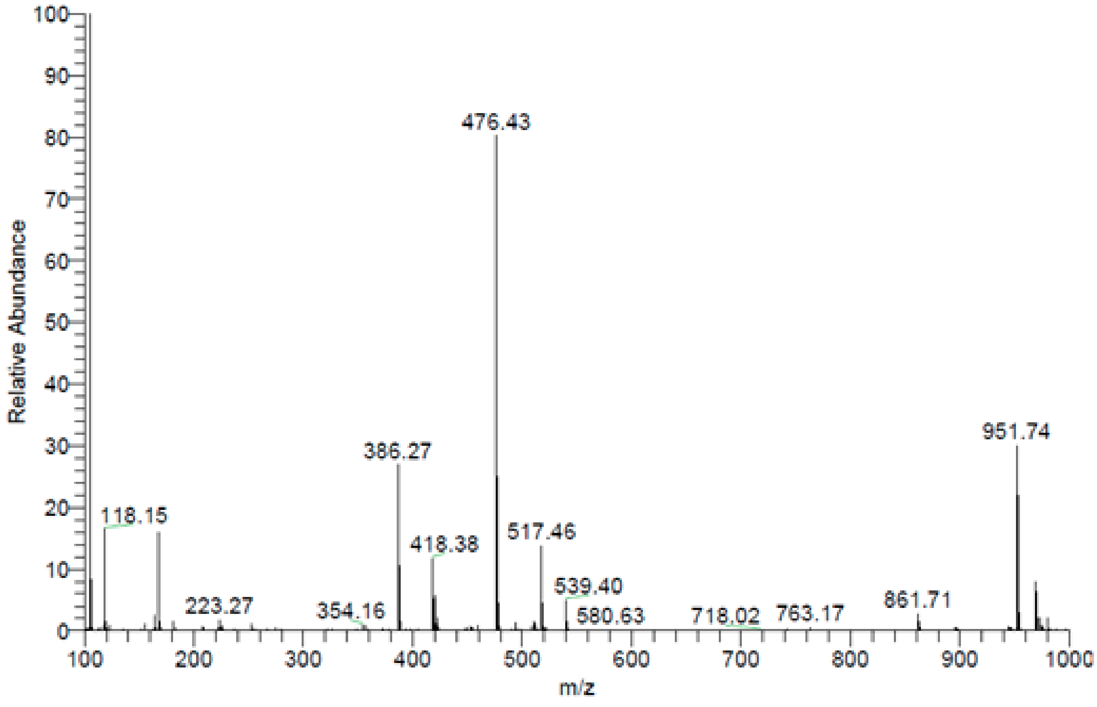

Figure S26. Mass spectrum of compound (**4e**)

***5-(2-benzoyloxybenzoyl)*-3-(4-chlorophenyl*)-4-phenyl isoxazole* (4f):**

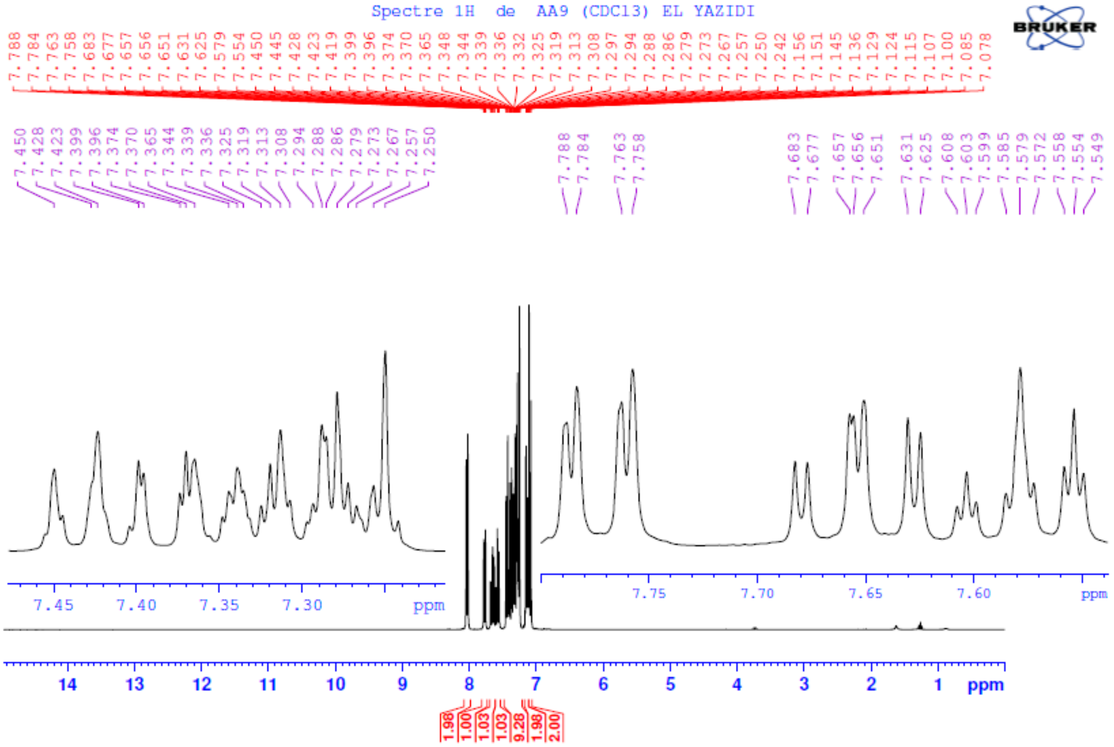

Figure S27. ^{1}H NMR spectrum (300 MHz, $CDCl_3$) of compound (**4f**)

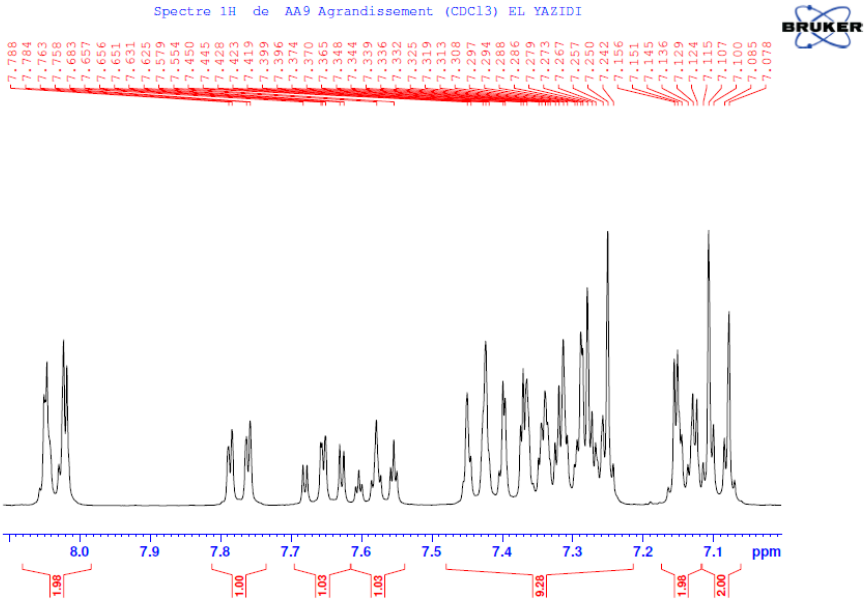

Figure S28. Aromatic enlarged region of ^{1}H NMR spectrum of compound (**4f**)

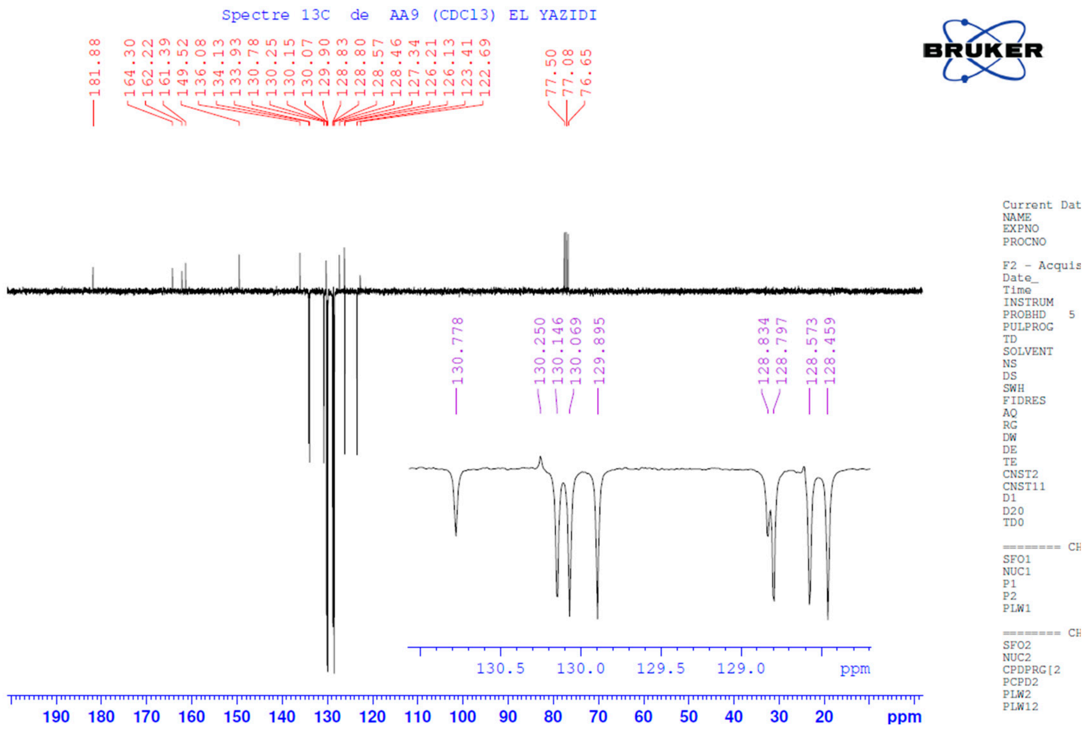

Figure S29. ^{13}C NMR spectrum (75 MHz, $CDCl_3$) of compound (**4f**)

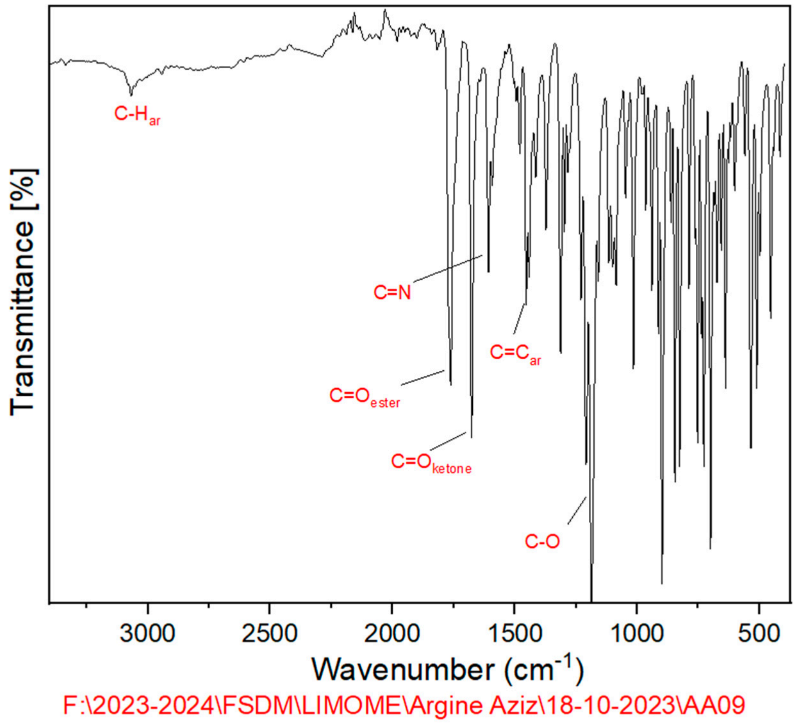

Figure S30. IF spectrum of compound (**4f**)

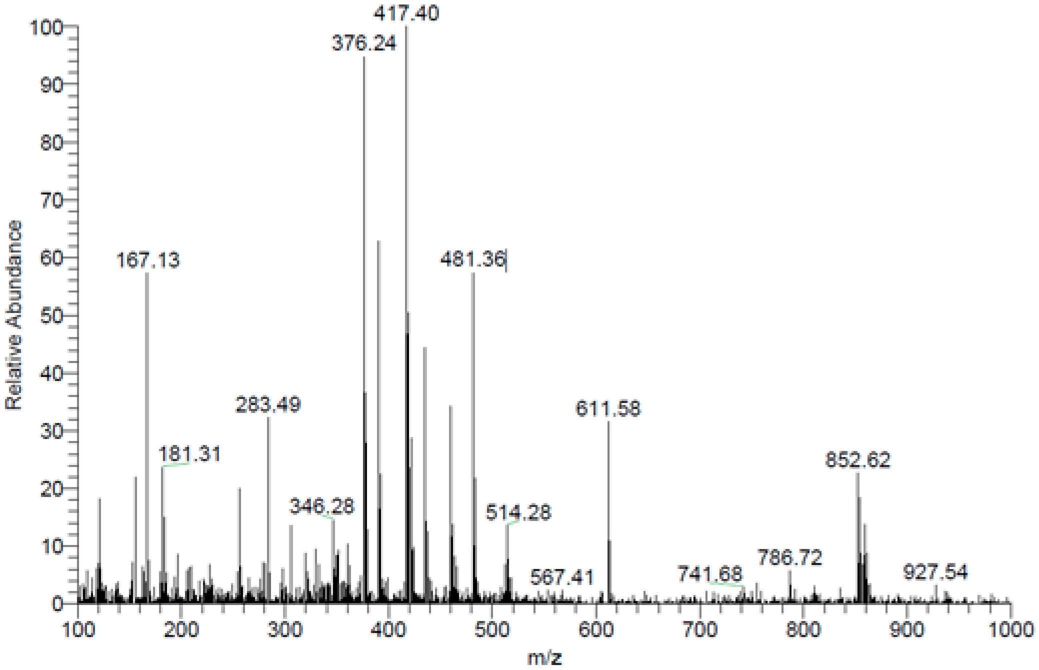

Figure S31. Mass spectrum of compound (**4f**)

References

1. Beney, C.; Mariotte, A.; Boumendjel, A. An Efficient Synthesis of 4 ,6 Dimethoxyaurones. *Heterocycles* **2001**, *55*, 967–972, doi:10.3987/COM-01-9182.
2. Masoomi, S.; Alipour, E.; Ali, M.; Reza, A.; Shafiee, A. Synthesis of Novel 2-Benzylidenebenzofuran-3(2*H*)-One Derivatives. *Iran. J. Org. Chem.* **2011**, *3*, 733–736.
3. Arzine, A.; Abchir, O.; Chalkha, M.; Chebbac, K.; Rhazi, Y.; Barghady, N.; Yamari, I.; Moussaoui, A.E.L.; Nakkabi, A.; Akhazzane, M.; et al. Design, Synthesis, In-Vitro, In-Silico and DFT Studies of Novel Functionalized Isoxazoles as Antibacterial and Antioxidant Agents. *Comput. Biol. Chem.* **2023**, 107993, doi:10.1016/j.compbiolchem.2023.107993.
4. *Bruker APEX4, SAINT & SHELXTL*; Bruker AXS LLC: Madison, Wisconsin, USA, 2021.
5. Krause, L.; Herbst-Irmer, R.; Sheldrick, G.M.; Stalke, D. Comparison of Silver and Molybdenum Microfocus X-Ray Sources for Single-Crystal Structure Determination. *J. Appl. Crystallogr.* **2015**, *48*, 3–10, doi:10.1107/S1600576714022985.
6. Sheldrick, G.M. SHELXT - Integrated Space-Group and Crystal-Structure Determination. *Acta Crystallogr. Sect. A Found. Crystallogr.* **2015**, *71*, 3–8, doi:10.1107/S2053273314026370.
7. Sheldrick, G.M. Crystal Structure Refinement with SHELXL. *Acta Crystallogr. Sect. C Struct. Chem.* **2015**, *71*, 3–8, doi:10.1107/S2053229614024218.
8. Brandenburg, K.; Putz, H. *DIAMOND*; Crystal Impact GbR: Bonn, Germany, 2012.
9. Chalkha, M.; Nour, H.; Chebbac, K.; Nakkabi, A.; Bahsis, L.; Bakhouch, M.; Akhazzane, M.; Bourass, M.; Chtita, S.; Bin Jardan, Y.A.; et al. Synthesis, Characterization, DFT Mechanistic Study, Antimicrobial Activity, Molecular Modeling, and ADMET Properties of Novel Pyrazole-Isoxazoline Hybrids. *ACS Omega* **2022**, *7*, 46731–46744, doi:10.1021/acsomega.2c05788.
10. Chebbac, K.; Benziane Ouaritini, Z.; El Moussaoui, A.; Chalkha, M.; Lafraxo, S.; Bin Jardan, Y.A.; Nafidi, H.A.; Bourhia, M.; Guemmouh, R. Antimicrobial and Antioxidant Properties of Chemically Analyzed Essential Oil of *Artemisia Annua* L. (Asteraceae) Native to Mediterranean Area. *Life* **2023**, *13*, 807, doi:10.3390/life13030807.
